# Synthesis and Preliminary Anticancer Activity Assessment of N-Glycosides of 2-Amino-1,3,4-thiadiazoles

**DOI:** 10.3390/molecules26237245

**Published:** 2021-11-29

**Authors:** Katarzyna Żurawska, Marcin Stokowy, Patryk Kapica, Monika Olesiejuk, Agnieszka Kudelko, Katarzyna Papaj, Magdalena Skonieczna, Wiesław Szeja, Krzysztof Walczak, Anna Kasprzycka

**Affiliations:** 1 Department of Organic Chemistry, Bioorganic Chemistry and Biotechnology, Faculty of Chemistry, Silesian University of Technology, Krzywoustego Street 4, 44-100 Gliwice, Poland; Katarzyna.Hopko@polsl.pl (K.Ż.); szoko1@gmail.com (M.S.); kapica.patrick@gmail.com (P.K.); wieslaw.szeja@adres.pl (W.S.); krzysztof.walczak@polsl.pl (K.W.); 2 Centre of Biotechnology, Silesian University of Technology, Krzywoustego Street 8, 44-100 Gliwice, Poland; katarzyna.papaj@polsl.pl (K.P.); Magdalena.Skonieczna@polsl.pl (M.S.); 3 Department of Chemical Organic Technology and Petrochemistry, The Silesian University of Technology, Krzywoustego Street 4, 44-100 Gliwice, Poland; monika.olesiejuk@polsl.pl (M.O.); Agnieszka.Kudelko@polsl.pl (A.K.); 4 Department of Systems Biology and Engineering, The Silesian University of Technology, Akademicka Street 16, 44-100 Gliwice, Poland

**Keywords:** glycal, N-glycosides, molecular iodine, 1,3,4-thiadiazole

## Abstract

The addition of 2-amino-1,3,4-thiadiazole derivatives with parallel iodination of differently protected glycals has been achieved using a double molar excess of molecular iodine under mild conditions. The corresponding thiadiazole derivatives of N-glycosides were obtained in good yields and anomeric selectivity. The usage of iodine as a catalyst makes this method easy, inexpensive, and successfully useable in reactions with sugars. Thiadiazole derivatives were tested in a panel of three tumor cell lines, MCF-7, HCT116, and HeLa. These compounds initiated biological response in investigated tumor models in a different rate. The MCF-7 is resistant to the tested compounds, and the cytometry assay indicated low increase in cell numbers in the sub- G1 phase. The most sensitive are HCT-116 and HeLa cells. The thiadiazole derivatives have a pro-apoptotic effect on HCT-116 cells. In the case of the HeLa cells, an increase in the number of cells in the sub-G1- phase and the induction of apoptosis was observed.

## 1. Introduction

1,3,4-Thiadiazole represents an important heterocyclic scaffold due to its pharmacological activity. Their derivatives exhibit various and significant activities such as antibacterial [1], antifungal, anti-inflammatory [2], anti-tuberculosis [3], antidepressant [4], and anticancer [5,6,7] activities, etc. The 1,3,4-Thiadiazole ring influences cancer cell lines mainly due to ability of nitrogen atoms to donate electrons and form hydrogen bonds or chelate certain metal ions [8]. The introduction of additional substituents (e.g., the -NH_2_, SO_3_H group, etc.) on the C-2 and/or C-5 carbon atoms of the thiadiazole ring enhances the desired properties. Moreover, the amino derivatives of 1,3,4-thiadiazole, which have different biocidal effects, are also investigated, probably due to the toxophore group N=C-S- [8,9,10]. As ligands, they also provide many potential binding sites for complexation and have diverse biological activity [11].

**Figure 1:**
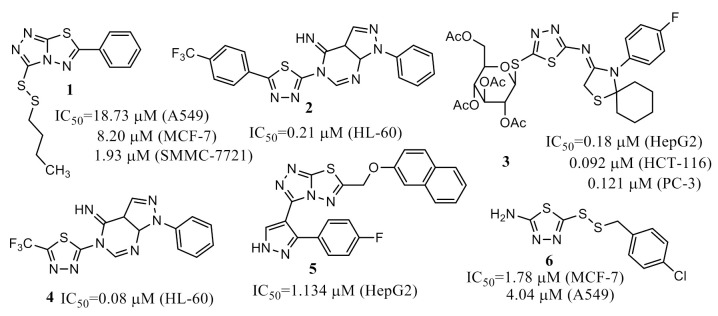
Anti-tumor active compounds with 1,3,4-thiadiazole core [12].

It is well known that derivatives of 1,3,4-thiadiazoles exhibit anti-tumor activity against human cancer cells (MCF-7 (breast cancer), SMMC-7721 (human hepatocarcinoma), HL-60 (human leukemia), A549 (non-small cell lung carcinoma), Hep-G2 (human liver hepatocellular carcinoma), PC-3 (human prostate adenocarcinoma), and HCT-116 (human colorectal carcinoma); Figure 1) [12,13,14,15,16,17,18]. The molecular targets belong to a group of proteins involved in proliferation, survival, and metastasis. These include carbonic anhydrase, matrix metalloproteinases, histone deacetylases, tubulin, focal adhesion kinases, protein tyrosine kinases, etc. [8,9,19,20,21,22,23,24,25,26,27,28]. The conjugation of drugs with sugar molecules in many cases improves their distribution into the cells. Several anticancer drugs such as glufosfamide, paclitaxel, and chlorambucil were conjugated with glucose for improving cancer targeting and selectivity [29].

Priebe et al. [30] reported the synthesis of new iodo-hexose compounds and their preliminary antitumor application for the treatment of glioblastoma and pancreatic cancer, which showed better anti-tumor activity in contrast to their deoxy-analogs. On the other hand, glycals are versatile building blocks in carbohydrate synthetic chemistry and have been extensively investigated for the synthesis of 2-deoxy-*O*-, *S*-, *N*-glycosides. The method for entry into 2-deoxy glycosides has been discussed in several reviews [31,32,33,34,35].

Continuing this study, we present the synthesis and biological activity of conjugates possessing different 2-deoxy-2-iodomonosaccharide scaffolds joined with thiadiazole derivatives.

## 2. Results and Discussion

### 2.1. Synthesis

**Scheme 1:**
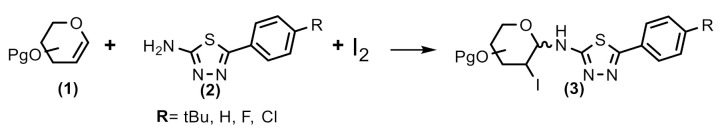
Synthesis of glycoconjugates, derivatives of 1,2,4-thiadiazole.

In the present work, the synthesis of aminoglycosides in the reaction of glycals with 2-amino-1,3,4-thiadiazole derivatives has been studied. The preliminary antitumor activity of obtained conjugates was determined. The targeted conjugates were obtained by the addition of the 2-amino-1,3,4-thiadiazole derivative (**2**) to an unsaturated sugar (**1**) in the presence of iodine (Scheme 1).

The glycosylation of the glycal derivatives was performed in the dark at room temperature. The main products formed by the addition of iodine (Scheme 1) to the double bond of 3,4-di-*O*-acetyl-L-rhamnal (**1a**), 3,4-di-*O*-benzyl-L-rhamnal (**1d**), 3,4,6-tri-*O*-acetyl-D-glucal (**1b**), and 3,4,6-tri-*O*-acetyl-D-galactal (**1c**), followed by a substitution reaction, were isolated by column chromatography. The structures of the obtained compounds were established based on the analysis of their NMR spectra. The signals were identified using literature data [36,37]. Additionally, in the ^1^H NMR spectra, the following were diagnostic: chemical shifts of acetyl groups, hydrogen at the anomeric position, hydrogens at C-6 position for rhamnopyranose derivatives, and hydrogen at C-2. In the ^13^C NMR spectrum were the C-1, C-2, and S-C=N carbon chemical shifts.

To determine the reaction conditions, we performed preliminary experiments presented in: Table 1.

When the 3,4-di-*O*-acetyl-L-rhamnal (**1a**) was treated with 2-amino-5-(4-*tert*-butylphenyl)-1,3,4-thiadiazole (**2a**) in different solvents (**Entry a**–**c**) and in the absence of iodine, no substrate conversion was observed. In the second set of experiments, the reaction was carried out in the presence of 1 eq. of iodine in the mixture of solvents with different polarities (**Entry d**–**f**). Under applied conditions, only traces of the product were detected. The third set of experiments was carried out in THF in the presence of a different concentration of iodine, namely, 0.5, 1.0, and 2.0 eq (**Entry g**–**i**). These results indicated that the reaction occurred successfully in the presence of double molar excess of molecular iodine. To determine which of the iodoamination methods would be the most beneficial, an experiment with NIS was also performed (**Entry j**), this reactant is recommended as conventional in synthesis 2-deoxy-2-halogenosugars starting from glycals [36]. The presence of an additional product in a very high concentration prompted us to abandon this method. Other methods of addition using heavy metals as catalysts are also known [38]. Unfortunately, the residue of those metals in the tested sample could adversely affect the survival of cells and the results of biological tests, so we decided to give up these methods as well.

In a series of experiments, we carried out reactions of 3,4-di-*O*-acetyl-L-rhamnal (**1a**), 3,4,6-tri-*O*-acetyl-D-glucal (**1b**), 3,4,6-tri-*O*-acetyl-D-galactal (**1c**), and 3,4-di-O-benzyl-L-rhamnal (**1d**) with 2-amino-5-phenyl-1,3,4-thiadiazole derivatives (**2a**–**2d**) containing different substituents in the aromatic ring in anhydrous THF containing molecular iodine. As a result of these experiments, hitherto unknown derivatives **3a**–**3m** were obtained. The results are presented in Table 2.

#### Proposed Mechanism

The anomeric configurations of all products were established based on their ^1^H and ^13^C NMR spectra analysis. The main products (**3a**, **3b**, **3c**, **3d**) obtained from L-rhamnal diacetate (**1a**) showed large values of the coupling constants (for isomer β: *J*_1,2_ (9.6–10.4 Hz), *J*_2,3_ (9.6 Hz), and *J*_3,4_ (9.6 Hz)), indicating the trans-diaxial disposition between H-1 and H-2, H-2 and H-3, and H-3 and H-4 in the favored ^5^H_4_ conformation. Large *J*_1,2_ values in compounds **3a**–**3d** signify the L-gluco configuration. The reaction of 3,4-di-*O*-benzyl-L-rhamnal (**1d**) with 2-amino-5-phenyl-1,3,4-thiadiazole (**2b**) led to 1:1 α/β mixture of L-gluco stereoisomers.

Iodine-promoted iodoamination of d-glucal (**1b**) with 2-amino-5-phenyl substituted 1,3,4-thiadiazole derivatives (**2a** and **2d**) was highly stereoselective, affording the β-1,2-trans isomer of D-glucose (**3e** and **3h**) in moderate yields (Table 2, **Entry e** and **h**). Under the same conditions, D-galactal (**1c**) was transformed into an inseparable mixture of 2-deoxy-2-iodo-α/β-galacto N-glycosides (Table 2, **Entry i, j, k,** and **l**) in modest 45%–72% overall yields (Table 2). These last compounds were identified from the ^1^H NMR proton coupling constants (for isomer α: *J*_1,2_ = 7.8–8.8 Hz, *J*_2,3_ = 3.0–3.6 Hz, *J*_3,4_ = 5.4–6.0 Hz, and for isomer β: *J*_1,2_ = 8.4–10.8 Hz, *J*_2,3_ = 5.6 Hz, *J*_3,4_ = 3.2 Hz).

The electrophilic addition of halogens to cyclic enol ethers has been investigated earlier by Lemieux and Fraser-Reid [39], who proposed a general mechanism involving the initial formation of carbenium ions which, upon nucleophilic attack by halide ion, give mainly the products of thermodynamic control. Later on, it has been shown that the product formation is under kinetic control and that the stereoselectivity depends on the solvent polarity [7,9,10], the structure of the enol ether, and the nature of the halogen [16,40,41,42].

To improve the stereoselectivity of glycosylation reactions involving glycals, directing groups such as Cl, Br, and I are introduced. Most commonly, a directing group is temporarily introduced at C-2, and the acceptor is then incorporated at C-1. An example is NIS- or NBS-mediated glycosylation developed by Thiem et al. These protocols have found widespread use in the construction of complex amine-containing N-glycosides [39,43].

**Scheme 2:**
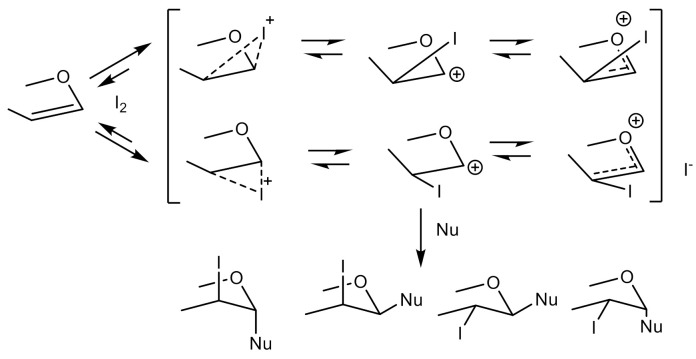
Addition of iodine to the double bond of glycals followed glycosylation.

The addition of halogen to double bonds is expected to occur by a bimolecular process, with the approach of a halogen molecule perpendicularly to the system of the vinyl ether from both site of a molecule plane, and the compound formed may rearrange in the rate-limiting step to intermediate ions (ion pairs, open carbocation, or carboxonium ion) which, according to their relative reactivities, control the stereochemical course of the reaction (Scheme 2).

Unsaturated pyranoid rings are conformationally more flexible than saturated ones. Therefore, even insignificant changes to the spatial disposition of substituents attached to the ring may give rise to changes in the ^4^H_5_, ^5^H_4_ conformational equilibrium of glycals. The factor affecting the conformational equilibrium of glycals is the 1,3-diaxial interactions destabilizes the ^5^H_4_ form of D-glycals with the C-3 and C-5 substituents in the cis orientation [44].

Compounds **1a** exist mainly in the ^5^H_4_ conformation (Scheme 2), where all of the substituents have quasiequatorial orientations. In the ^4^H_5_, the acetoxyl group at C-4 is in the quasiaxial disposition, and this destabilizes this structure.

**Scheme 3:**
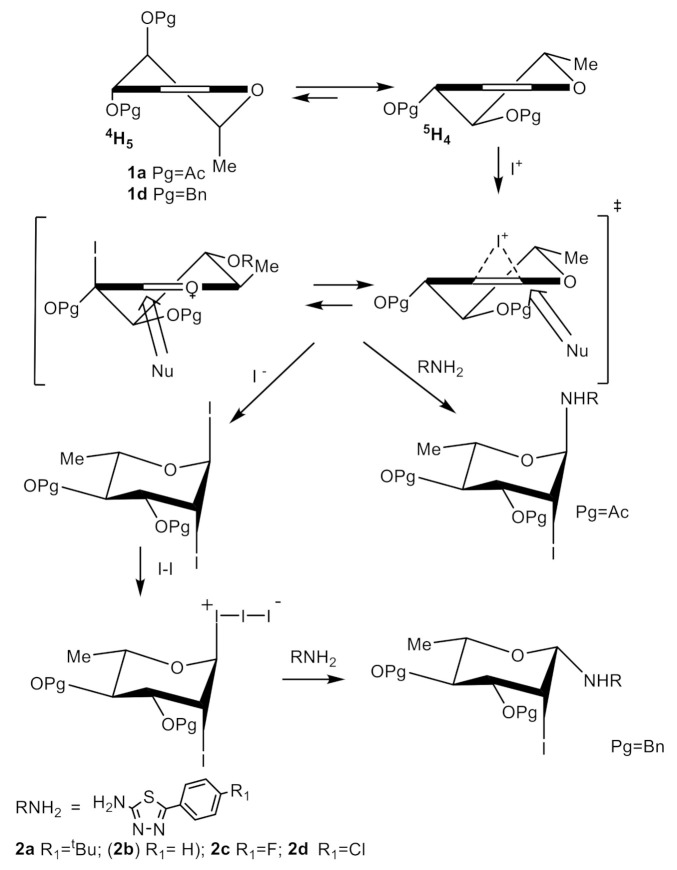
Synthesis of 2-amino-1,3,4-thiadiazole glycosides starting from L-rhamnal (**1a**) with stereochemical effects.

Stereoselectivity of the iodonium-promoted electrophile addition to glycals is controlled, among others, by the preferred initial attack on the glycal. It is well accepted that iodine-based electrophiles preferentially react from the top-face of glycals in their half-chair more stable conformation (Scheme 3) and the formation of bridged iodonium intermediate **1a**–**d**, whose further trapping by nucleophiles can easily account for the regiospecificity and the high trans stereoselectivity observed [17,45,46,47,48,49,50,51,52,53,54,55,56,57].

The stereoselectivity of reaction depends on the structure of protecting groups. In the reaction of 3,4-di–*O*-benzyl-L-rhamnal (**1d**) a mixture of 1,2-trans L-gluco and L-manno glycosides was formed, suggesting that the addition of iodine proceeds through the stabilized carbo-oxonium ion (Scheme 3). It is proposed that the stereoselectivity of the reaction is based on a Curtin–Hammett kinetic scheme involving the in situ generation of the more reactive glycosyl halide [58].

The 1,2-trans diiodie is formed by preferential attack of I^-^ on the oxocarbenium ion and the next activation in reaction with iodine takes place. The isomerization of iodides is a fast reaction, and an equilibrium mixture of α,β-iodides is formed [59].

The 1,2-cis glycoside is formed by attack of the nucleophile on the more reactive 1,2-trans-di-iodide via an SN2-like substitution (Scheme 3) and gives 1,2-cis glycoside as the main product of the reaction. The 1,2-trans isomer can alternatively be received by the competitive formation of 2-iodo-α-glycosyl iodide, which could then give a N-glycoside in direct SN2 substitution with the amine. On the other hand, the 1,2-trans glycoside may be obtained in the reaction of amines with carbooxonium ion.

**Scheme 4:**
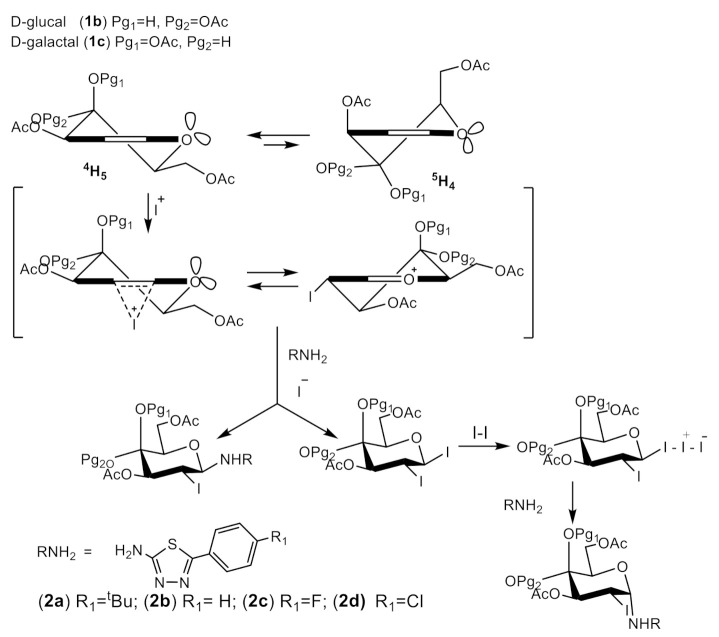
Synthesis of 1,3,4-thiadiazole derivative glycosides starting from D-glucal (**1b**) and D-galactal (**1c**) with stereochemical effects.

Tri-*O*-acetyl-D-glucal (**1b**) exists mainly in the ^5^H_4_ conformation (Scheme 4), and all of the substituents in **1b** have quasiequatorial orientations. The main product, as in the bromination of glucal in Priebe et al.’s experiments [59], was compound having the α-D-manno configuration. This result may be explained by the formation of bridged iodonium ions that induce the attack of iodide from the opposite side (trans opening), affording the production of the trans configuration (β-gluco iodide). The fast equilibration leads to the mixture of α/β iodides, and the nucleophilic substitution of the anomeric carbon atom by attack of 4-chloro or 5-tertbutyl substituted derivatives is controlled by steric effects. The preferred reaction by equatorial attack leads to cis β-manno glycoside formation [60].

The results of galactal **1c** are opposite to the results presented above. The main products are 1,2-cis glycosides. The overall results were very close to those for the bromination of tri-*O*-acetyl-D-galactal, indicating that electronic effects rather than purely steric effects of the C-6 substituent are responsible for the product distribution during the bromination of glycals [15,16,17], where galactals with electron-withdrawing substituents at C-6 gave ~90% of the trans addition products [16]. The approach of iodine from above the molecular plane leads to the β-galacto isomer and is energetically more favorable than when the attack is from below the plane of the molecule. Due to steric reasons, the S_N_2 substitution of I_3_ led to the α-D-galacto isomer.

### 2.2. Biological Evaluation and Anticancer Screening

#### 2.2.1. Viability of Cancer Cell Lines

**Figure 2:**
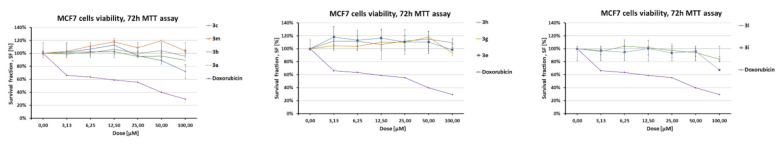
SF of MCF-7 cells after 72 h of incubation with different doses of the tested compounds (0–100 µM) and doxorubicin as a positive control. The results were presented as the mean values from 3 experiments ± SD.

**Figure 3:**
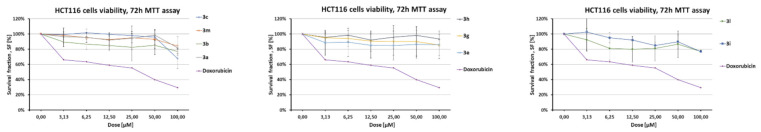
SF of HCT116 cells after 72 h of incubation with different doses of the tested compounds (0–100 µM) and doxorubicin as a positive control. The results were presented as the mean values from 3 experiments ± SD.

**Figure 4:**
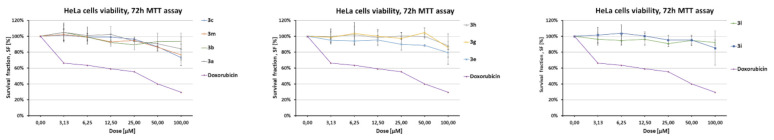
SF of HeLa cells after 72 h of incubation with different doses of tested compounds (0–100 µM) and doxorubicin as a positive control. The results were presented as the mean values from 3 experiments ± SD.

The influences of the compounds on three types of cancer cell lines (i.e., MCF-7, HCT116, and HeLa) were determined with MTT assay. After a 72 h incubation with the tested compounds, the cells’ viabilities were measured and presented as Survival Fraction s(SF) in Figure 2, Figure 3 and Figure 4.

The results from the MTT assay for MCF-7 cells did not allow the IC_50_ calculation. However, in the case of two compounds (**3c** and **3j**), the SF of the cells decreased significantly at the highest doses (Figure 2).

Based on the results obtained for the HCT116 cell line, we concluded that this cell line was more sensitive to the tested compounds than the MCF-7 cell line, as can be seen in Figure 3.

In the case of HeLa cells, the effect of the compounds on the SF value was visible for most of the compounds, but it was not as strong as in the case of HCT116 cells (Figure 4).

#### 2.2.2. Cytostatic Effects on Cancer Cell Lines followed by Cell Cycle

Although the MTT assay did not deliver clear results for the cytotoxicity of the tested compounds, the effect of lowered viabilities may have been caused by the cell cycle arrest. For a better interpretation of the possible effects, we decided to perform flow cytometry analysis for the highest doses of the compounds (100 µM). The typical histograms of DNA content in cells are presented in Supplementary materials (Figure S1).

**Figure 5:**
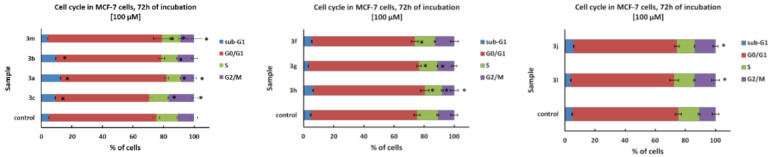
Cell cycle in MCF-7 cells after a 72 h incubation with compounds with the concentration of 100 µM. The results were presented as mean value ± SD. The statistical analysis was based on a t-test, and a *p*-value less than 0.05 was considered statistically significant in comparison to the untreated controls (indicated with a star).

In the case of MCF-7 cells, some of the tested compounds (**3a** and **3c**) influenced the cell cycle distribution and increased the number of cells in the sub-G1 phase, which corresponds to the dead cells (Figure 5). Furthermore, we observed a visible cell cycle arrest in the G0/G1 phase after the exposition of the cells on the compounds **3f**, **3g,** and **3h**.

**Figure 6:**
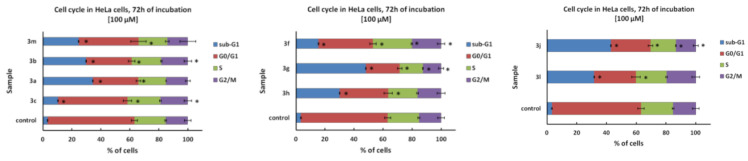
Cell cycle in HCT116 cells after a 72 h incubation with compounds with the concentration of 100 µM. The results were presented as mean value ± SD. The statistical analysis was based on a t-test, and a *p*-value less than 0.05 was considered statistically significant in comparison to the untreated controls Table 3 (indicated with a star).

Cells responded with more visible cytostatic and cytotoxic effects (Figure 6). For a panel of **3a**, **3b**, **3c**, and **3m** compounds, the number of cells in the sub-G1 phase increased. Additionally, the lowering of the number of cells in the S phase and cell cycle arrest in the G2/M phase indicated the DNA damage with inhibition of the replication process.

**Figure 7:**
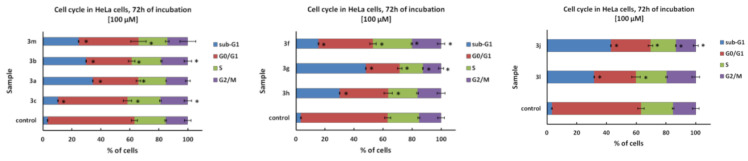
Cell cycle in HeLa cells after a 72 h incubation with compounds with the concentration of 100 µM. The results were presented as mean value ± SD. The statistical analysis was based on a t-test, and a *p*-value less than 0.05 was considered statistically significant in comparison to the untreated controls (indicated with a star).

In the case of the HeLa cell line, we observed a significant increase in the number of cells in the sub-G1 phase, which suggests lethal effects of the tested compounds on this cell line (Figure 7).

We decided to further investigate the type of cell death for each cell line with Annexin V with PI assay, which allows to distinguish necrosis from apoptosis.

#### 2.2.3. Death Mechanism Induced in Cancer Lines

To conclude the cell death mechanism, we performed Annexin V with PI assay after a 72 h incubation of all the cell lines with the analyzed compounds in the concentration of 100 µM. The typical dot plots from Annexin-V apoptosis assay from cells are presented in Supplementary material (Figure S2).

**Figure 8:**
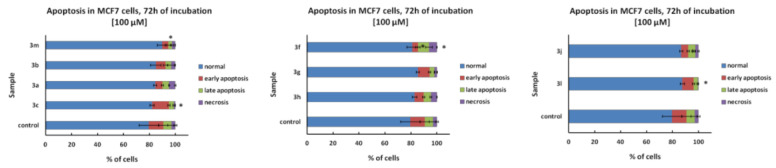
Apoptosis in MCF-7 cells after a 72 h incubation with compounds with a concentration of 100 µM. The results were presented as mean value ± SD. The statistical analysis was based on a t-test, and a *p*-value less than 0.05 was considered statistically significant in comparison to the untreated controls (indicated with a star).

Based on the results, for the MCF-7 cell line (Figure 8), we concluded that the tested compounds did not exhibit any pro-apoptotic effects.

**Figure 9:**
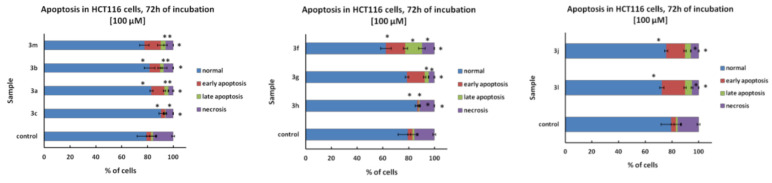
Apoptosis in HCT116 after a 72 h incubation with compounds with a concentration of 100 µM. The results were presented as mean value ± SD. The statistical analysis was based on a t-test, and a *p*-value less than 0.05 was considered statistically significant in comparison to the untreated controls (indicated with a star).

In contrast to the MCF-7, the HCT116 cells (Figure 9)responded with apoptosis. It indicated that the analyzed compounds exhibit pro-apoptotic activity against this cell line.

**Figure 10:**
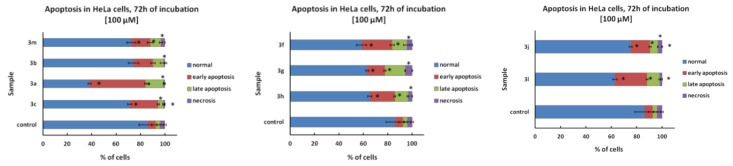
Apoptosis in HeLa after a 72 h incubation with compounds with the concentration of 100 µM. The results were presented as mean value ± SD. The statistical analysis was based on a t-test, and a p-value less than 0.05 was considered statistically significant in comparison to the untreated controls (indicated with a star).

Similarly to the HCT116 cells, the tested compounds have exhibited a pro-apoptotic effect on the HeLa cell line. However, in most of the cases, the effect was much stronger (Figure 10).

The efficient formation of glycosidic linkages has played an important role in the development of modern synthetic carbohydrate chemistry. Among the various types of donors that can be employed for the construction of glycosidic bonds, glycosyl iodides should now be viewed as versatile glycosylation reagents [61,62].

The research found the ineffectiveness of the conventional methods for the preparation of N-glycosides of 2-deoxy monosaccharides in the nucleophilic substitution reaction with the participation of very weakly nucleophilic amine thiadiazole derivatives. The publication presents an efficient method for glycosylation starting from glycals, with the activation leading to the glycosyl donor taking place with 2eq of iodine, preferably in a polar solvent such as THF. It has been shown that by the appropriate optimization of conditions, it is possible to obtain highly stereoselective products of rhamnal and glucal. The advantage of this method is the use of an easily available, cheap, and environmentally friendly reagent, molecular iodine.

The biological activity of the tested compounds was determined based on the MTT assay complemented with flow cytometry analysis. The combination of these methods allows for the detection of cytotoxic, cytostatic, and lethal effects of the compounds on the analyzed cell lines.

The performed MTT assay indicated that the effect of the compounds depended significantly on the tested cell line (Table 3). Among the tested cell lines, MCF-7 cells were the most resistant and HCT116 cells were the most sensitive to the tested compounds. Unfortunately, the compounds exhibited low biological activity and hence neither the IC_50_ parameter nor the selectiveness of the compounds against the tested cell lines could be evaluated for the examined doses. Nevertheless, based on the SF values, we observed that the highest concentrations of the compounds (100 µM) inhibited the cells proliferation. Because of it, we decided to test the effect of the compounds on the cell cycle only for the highest concentrations.

According to the biological assays, in the case of MCF-7 cells, despite the low cytotoxic effect of the compounds, we did not observe any cytostatic or pro-apoptotic effect. During the cell cycle analysis, we detected a slight increase in the number of cells present in the sub-G1 phase or an increase in cells arrested in the G0/G1 phase. Furthermore, there was no increase in apoptotic nor necrotic cells. Nevertheless, the detected effect was not significant when compared to the untreated control.

More promising effects were detected in the case of other cell lines. The cell line, which was the most sensitive in the MTT assay (HCT116) responded with both cytostatic and cytotoxic effects. During the cell cycle analysis, we observed an increase in the number of cells in the sub-G1 fractions, however, the strongest effect was visible in the inhibition of the S phase and the arrest of the cells in the G2/M phase. Additionally, we detected an increase in cells in the early apoptosis, which suggests that the compounds have pro-apoptotic activity against the HCT116 cell line.

The most auspicious effects were observed for the HeLa cells. Similarly to the HCT116 cell line, the compounds caused cytotoxic and cytostatic effects on this cell line. Moreover, in the case of the HeLa cells, we detected an increase in the number of cells in the sub-G1 phase. Furthermore, Annexin V staining proved that the cells underwent both apoptotic cell deaths. 

Based on the carried out experiments we noticed a tissue-dependent effect of the tested compounds and we concluded that they exhibit a cytotoxic effect on the MCF-7, HCT116, and HeLa cell lines, a cytostatic effect on the HCT116 and HeLa cell lines, and a lethal effect on the HeLa cell line.

Our results indicate that the tested compounds in their current form cannot be exploited as drugs, however, they stand as promising models for further modification. In order to improve the antitumor activity of the conjugates, structural modifications are necessary. Especially interesting is applying in biological tests deprotected conjugates, it should affect the solubility of these derivatives in the applied medium. Another approach is the synthesis of the derivatives where a sugar moiety is linked to a thiadiazole derivative via an aliphatic, aromatic-aliphatic, or aromatic linker.

## 3. Materials and Methods

### 3.1. Chemistry

General. All reagents were purchased from Merck (Darmstadt, Germany), Acros Organics (Geel, Belgium), Alpha Aesar (Haverhill, USA), and Sigma-Aldrich (Taufkirchen, Germany) and used without further purification. A column chromatography was per-formed on a silica gel packed column (Kieselgel 60 0.040–0.063 mm, 230–400 mesh, Merck, Darmstadt, Germany). Solvents for chromatography (n-hexane, ethyl acetate, methanol, dichloromethane) were distilled before use. Thin layer chromatography was performed on plates coated with silica gel 60 F_254_ (Merck, Darmstadt, Germany); detection was carried out with either ultraviolet light (254 nm) or spraying with a solution of phosphomolybdic acid, a basic potassium permanganate solution, or ethanolic solution of concentrated H_2_SO_4_, with subsequent heating. Optical rotations were measured with a JASCO P-2000 polarimeter using a sodium lamp (589.3 nm) at room temperature. Melting points were measured on a Stuart SMP3 melting point apparatus. The ^1^H NMR and ^13^C NMR spectra were recorded on an Agilent 400 MHz spectrometer and Varian 600 MHz spectrometer in DMSO-*d_6_* or CDCl_3_ using tetramethylsilane (TMS) as an internal standard. Chemical shifts are reported as *δ* values (ppm). FT-IR spectra were recorded between 4000 and 650 cm^−1^ using an FT-IR Nicolet 6700 apparatus (IET, Mundelein, USA) with a Smart iTR accessory. HRMS spectra were recorded on a Waters ACQUITY UPLC/Xevo G2QT instrument.

General procedure for iodination of glycals:

To a solution of glycal (**1a**–**d**: 1.0 eq) in THF (2 mL), 2-amino-1,3,4-thiadiazole derivative (**2a**–**d**: 2.0 eq) and I_2_ (2.0 eq) were added. The reaction was stirred at room temperature for 1.5h. The TLC (Toluene:EtOAc, 2:1 *v*/*v*) indicated total consumption of glycal. The solvent was removed under reduced pressure, and the residue was purified by silica gel column chromatography (gradient from toluene to toluene:EtOAc, 10:1 *v*/*v*) to give products as a pale yellow oil being a mixture of anomers. 

*5-(4-(tert-buthyl)phenyl)-N-(3,4-di-O-acetyl-2-deoxy-2-iodo-α/β-L-rhamnopyranosyl)-1,3,4-thiadiazol-2-amine* (**3a**): Starting from 3,4-di-*O*-acetyl-L-rhamnal (**1a**: 80 mg, 0.383 mmol) and 2-amino-5-(4-tert-buthylphenyl)-1,3,4-thiadiazole (**2a**: 174 mg, 0.746 mmol), yield 71% (62.9 mg); ratio of anomers (α:β = 1:11); ^1^H NMR (400 MHz, CDCl_3_): δ 7.58–7.56 (m, 4H, Ph), 7.46–7.43 (m, 4H, Ph), 6.04 (d, *J* = 4.6 Hz, 1H, H-1α), 5.90 (d, *J* = 10.4 Hz, 1H, H-1β), 5.44 (dd, *J* = 9.2 Hz, 11.2 Hz, 1H, H-4α), 4.87 (t, *J* = 9.6 Hz, 1H, H-3α), 4.69–4.62 (m, 1H, H-2α), 3.87 (dq, *J* = 6 Hz, 10 Hz, 1H, H-5α), 2.12 (s, 3H, COCH_3_), 2.08 (s, 3H, COCH_3_), 2.06 (s, 3H, COCH_3_), 2.05 (s, 3H, COCH_3_), 1.34 (s, 9H, C(CH_3_)_3_), 1.26 (d, *J* = 6 Hz, 1H, CH_3_α), and 1.19 (d, 1H, *J*= 6.4Hz, CH_3_β); ^13^C NMR (100 MHz, CDCl_3_): δ 170.0 (COCH_3_), 169.7 (COCH_3_), 167.2 (S-C=N), 154.4 (N-C=N), 127.4 (Ph), 126.2 (Ph), 126.0 (Ph), 81.6 (C-1), 76.3 (C-3), 74.0 (C-5), 72.7 (C-4), 35.1 (C(CH_3_)_3_), 31.3 (C(CH_3_)_3_), 29.8 (C-2), 21.0 (COCH_3_), 20.9 (COCH_3_), and 17.5 (C-6); HRMS *m/z* calcd for (C_22_H_28_IN_3_O_5_S + H^+^): 574.0867; found: 574.0874.

*5-phenyl-N-(3,4-di-O-acetyl-2-deoxy-2-iodo-α/β-L-rhamnopyranosyl)-1,3,4-thiadiazol-2-amine* (**3b**): Starting from 3,4-di-*O*-acetyl-L-rhamnal (**1a**: 220 mg, 1.027 mmol) and 2-amino-5-phenyl-1,3,4-thiadiazole (**2b**: 364 mg, 2.054 mmol), yield 47% (85.8 mg); ratio of anomers (α:β = 1:1.7); ^1^H NMR (600 MHz, CDCl_3_): δ 7.70–7.68 (m, 2H, Ph), 7.65–7.63 (m, 3H, Ph), 6.28 (d, *J* = 4.8 Hz, 1H, H-1α), 5.91 (d, *J* = 10.2 Hz, 1H, H-1β), 5.44 (dd, *J* = 9.0 Hz, 10.8 Hz, 1H, H-4β), 5.32 (dd, *J* = 3.6 Hz, 6Hz, 1H, H-3α), 5.10 (t, *J* = 4.8 Hz, 1H, H-2α), 5.04 (t, *J* = 6.6 Hz, 1H, H-4α), 4.88 (t, *J* = 9.6 Hz, 1H, H-3β), 4.69–4.62 (m, 1H, H-2β), 4.15–4.11 (m, 1H, 5α), 3.88 (dq, *J* = 6.6 Hz, 9.6 Hz, 1H, H-5β), 2.18 (s, 3H, COCH_3_), 2.12 (s, 3H, COCH_3_), 2.11 (s, 3H, COCH_3_), 2.06 (s, 3H, COCH_3_), 1.38 (d, *J* = 6.6 Hz, 1H, CH_3_α), and 1.28 (d, *J* = 6 Hz, 1H, CH_3_β); ^13^C NMR (150 MHz, CDCl_3_): δ 170.0 (COCH_3_), 169.8 (COCH_3_), 169.7 (COCH_3_), 162.1 (S-C=N), 147.4 (N-C=N), 130.8 (Ph), 130.2 (Ph), 129.1 (Ph), 129.0 (Ph), 128.3 (Ph), 126.4 (Ph), 126.4 (Ph), 76.3 (C-1), 74.0 (C-3), 72.8 (C-2), 72.0 (C-5), 71.3 (C-4), 26.8 (C-2), 21.2 (COCH_3_), 21.1 (COCH_3_), 21.0 (COCH_3_), 20.8 (COCH_3_), 17.5 (C-6), and 17.1 (C-6); HRMS *m/z* calcd for (C_18_H_20_IN_3_O_5_S + H^+^): 518.0241; found: 518.0251.

*5-(4-fluorophenyl)-N-(3,4-di-O-acetyl-2-deoxy-2-iodo-β-L-rhamnopyranosyl)-1,3,4-thiadiazol-2-amine* (**3c**): Starting from 3,4-di-*O*-acetyl-L-rhamnal (**1a**: 137 mg, 0.640 mmol) and 2-amino-5-(4-fluorophenyl)-1,3,4-thiadiazole (**2c**: 250 mg, 1.281 mmol), yield 55% (68.7 mg); ratio of anomers (α:β = 1:99); α^24^_D_ = -80 (c = 0.25, CHCl_3_); ^1^H NMR (600 MHz, CDCl_3_): δ 7.65–7.62 (m, 2H, Ph), 7.14–7.11 (m, 2H, Ph), 5.90 (d, 1H, *J* = 9.6 Hz, H-1β), 5.44 (dd, 1H, *J* = 9.6 Hz, 10.8 Hz, H-4β), 4.87 (dd, 1H, *J* = 9.6 Hz, H-3β), 4.67–4.60 (m, 1H, H-2β) 3.89–3.85 (m, 1H, H-5β), 2.12 (s, 3H, COCH_3_), 2.06 (s, 3H, COCH_3_), and 1.28 (d, 3H, *J* = 6Hz, CH_3_β); ^13^C NMR (150 MHz, CDCl_3_): δ 170.0 (COCH_3_), 169.7 (COCH_3_), 166.7 (S-C=N), 164.2 (d, *J* = 251 Hz, C-F), 154.8 (N-C=N), 132.4 (Ph), 128.4 (Ph), 128.3 (Ph), 116.3 (Ph), 116.2 (Ph), 83.8 (C-1), 76.3 (C-3), 74.0 (C-4), 72.8 (C-5), 27.0 (C-2), 21.0 (COCH_3_), 20.9 (COCH_3_), and 17.5 (C-6); HRMS *m/z* calcd for (C_18_H_19_FIN_3_O_5_S + H^+^): 536.0147; found: 536.0153.

*5-(4-chlorophenyl)-N-(3,4-di-O-acetyl-2-deoxy-2-iodo-β-L-rhamnopyranosyl)-1,3,4-thiadiazol-2-amine* (**3d**): Starting from 3,4-di-*O*-acetyl-L-rhamnal (**1a**: 121 mg, 0.565 mmol) and 2-amino-5-(4-chlorophenyl)-1,3,4-thiadiazole (**2d**: 239 mg, 1.129 mmol), yield 49% (58.6 mg); ratio of anomers (α:β = 1:99); α^24^_D_ = −13.3 (c = 0.45, CHCl_3_); ^1^H NMR (600 MHz, CDCl_3_): δ 7.58–7.56 (m, 2H, Ph), 7.42–7.40 (m, 2H, Ph), 5.91 (d, 1H, *J* = 9.6 Hz, H-1β), 5.44 (dd, 1H, *J* = 9.6 Hz, 11.4 Hz, H-4β), 4.87 (t, 1H, *J* = 9.6 Hz, H-3β), 4.65–4.59 (m, 1H, H-2β) 3.89–3.85 (m, 1H, H-5β), 2.12 (s, 3H, COCH_3_), 2.06 (s, 3H, COCH_3_), and 1.28 (d, 3H, *J* = 6Hz, CH_3_β); ^13^C NMR (150 MHz, CDCl_3_): δ 170.0 (COCH_3_), 169.7 (COCH_3_), 165.8 (S-C=N), 146.3 (N-C=N), 136.9 (C-Cl), 136.4 (Ph), 129.3 (Ph), 127.6 (Ph), 83.8 (C-1), 76.2 (C-3), 74.0 (C-4), 72.8 (C-5), 42.7 (C-2), 21.0 (COCH_3_), 20.9 (COCH_3_), and 17.5 (C-6); HRMS *m/z* calcd for (C_18_H_19_ClIN_3_O_5_S + H^+^): 551.9851; found: 551.9855.

*5-(4-(tert-buthyl)phenyl)-N-(3,4,6-tri-O-acetyl-2-deoxy-2-iodo-β-L-glucopyranosyl)-1,3,4-thiadiazol-2-amine* (**3e**): Starting from 3,4,6-tri-*O*-acetyl-D-glucal (**1b**: 360 mg, 1.322 mmol) and 2-amino-5-(4-tert-buthylphenyl)-1,3,4-thiadiazole (**2a**: 617 mg, 2.644 mmol), yield 51% (157.7 mg); ratio of anomers (α:β = 1:99); α^24^_D_ = 8.6 (c = 0.35, CHCl_3_); ^1^H NMR (400 MHz, CDCl_3_): δ 7.60–7.43 (m, 4H, Ph), 6.01 (d, *J* = 10.4 Hz, 1H, H-1β), 5.51 (dd, *J* = 8.8 Hz, 10.8 Hz, 1H, H-4β), 5.14 (dd, *J* = 9.6 Hz, 10 Hz, 1H, H-3β), 4.73–4.68 (m, 1H, H-2β), 4.35 (dd, *J* = 4.4 Hz, 12.8 Hz, 1H, H-6β), 4.15 (dd, *J* = 2.0 Hz, 12.4 Hz, 1H, H-6β), 4.10–4.06 (m, 1H, H-5β), 2.12 (s, 3H, COCH_3_), 2.06 (s, 3H, COCH_3_), 2.04 (s, 3H, COCH_3_), 1.35 (s, 3H, C(CH_3_)_3_), 1.34 (s, 3H, C(CH_3_)_3_), and 1.33 (s, 3H, C(CH_3_)_3_); ^13^C NMR (150 MHz, CDCl_3_): δ 170.5 (COCH_3_), 170.4 (COCH_3_), 170.1 (COCH_3_), 169.8 (COCH_3_), 165.5 (S-C=N), 156.2 (C-C(CH_3_)_3_), 154.4 (C-C(CH_3_)_3_), 146.2 (S-C=N), 129.3 (Ph), 128.1 (Ph), 127.0 (Ph), 126.8 (Ph), 98.6 (C-1), 80.2 (C-1), 74.2 (C-3), 73.2 (C-3), 70.5 (C-5), 68.2 (C-5), 63.5 (C-4), 63.2 (C-4), 62.2 (C-6), 60.8 (C-6), 36.1 (C-2), 31.5 (C(CH_3_)_3_), 29.6 (C(CH_3_)_3_), 27.3 (C-2), 21.9 (COCH_3_), 21.5 (COCH_3_), 20.9 (COCH_3_), 20.7 (COCH_3_), and 20.5 (COCH_3_); HRMS *m/z* calcd for (C_24_H_30_IN_3_O_7_S + H^+^): 632.09 22; found: 632.0930.

*5-phenyl-N-(3,4,6-tri-O-acetyl-2-deoxy-2-iodo-α/β-D-glucopyranosyl)-1,3,4-thiadiazol-2-amine* (**3f**): Starting from 3,4,6-tri-*O*-acetyl-D-glucal (**1b**: 243 mg, 0.893 mmol) and 2-amino-5-phenyl-1,3,4-thiadiazole (**2b**: 316 mg, 1.783 mmol), yield 68% (107.8 mg); ratio of anomers (α:β = 4.4:1); ^1^H NMR (600 MHz, CDCl_3_): δ 7.70–7.68 (m, 2H, Ph), 7.47–7.44 (m, 3H, Ph), 6.30 (d, *J* = 4.8 Hz, 1H, H-1β), 5.94 (d, *J* = 10.8 Hz, 1H, H-1α), 5.48 (dd, *J* = 9.6 Hz, 11.4 Hz, 1H, H-3α), 5.36 (dd, 1H, *J* = 4.2 Hz, 6.6 Hz, H-3β), 5.28 (t, *J* = 6.6 Hz, 1H, H-4β), 5.15–5.10 (m, 2H, H-4α, H-6β), 4.72–4.67 (m, 1H, H-6α), 4.37–4.34 (m, 2H, H-2α, H-6β), 4.29–4.22 (m, 2H, H-2β, H-5β), 4.14 (dd, *J* = 1.8 Hz, 12.6 Hz 1H, H-6α), 4.00 (m, 1H, H-5α), 2.19 (s, 3H, COCH_3_), 2.12 (s, 6H, COCH_3_), 2.09 (s, 3H, COCH_3_), and 2.07 (s, 3H, COCH_3_, 2.05 (s, 3H, COCH_3_); ^13^C NMR (150 MHz, CDCl_3_): δ 170.7 (COCH_3_), 169.5 (COCH_3_), 169.3 (COCH_3_), 161.5 (S-C=N), 147.5 (N-C=N), 130.8 (Ph), 129.0 (Ph), 126.2 (Ph), 82.9 (C-1), 72.9 (C-3), 70.8 (C-5), 67.5 (C-4), 61.6 (C-6), 25.7 (C-2), 21.0 (COCH_3_), 20.8 (COCH_3_), and 20.7 (COCH_3_); HRMS *m/z* calcd for (C_20_H_22_IN_3_O_7_S + H^+^): 576.0296; found: 576.0309.

*5-(4-fluorophenyl)-N-(3,4,6-tri-O-acetyl-2-deoxy-2-iodo-α/β-D-glucopyranosyl)-1,3,4-thiadiazol-2-amine* (**3g**): Starting from 3,4,6-tri-*O*-acetyl-D-glucal (**1b**: 304 mg, 1.117 mmol) and 2-amino-5-(4-fluorophenyl)-1,3,4-thiadiazole (**2c**: 436 mg, 2.233 mmol), yield 58% (126.4 mg); ratio of anomers (α:β = 1:1.3); ^1^H NMR (400 MHz, CDCl_3_): δ 8.15–8.10 (m, 2H, Ph), 7.16–7.12 (m, 2H, Ph), 6.27 (d, *J* = 4.8 Hz, 1H, H-1α), 5.99 (d, *J* = 10.4 Hz, 1H, H-1β), 5.50 (dd, *J* = 9.2 Hz, 11.2 Hz, 1H, H-3β), 5.33–5.30 (m, 1H, H-3α), 5.26 (t, *J* = 6.8 Hz, 1H, H-4α), 5.16–5.11 (m, 2H, H-4β, H-2α), 4.71–4.66 (m, 1H, H-2β), 4.44–4.21 (m, 4H, H-5α, H-6aα, H-6bα, H-6aβ), 4.14 (dd, *J* = 2.0 Hz, 14.4 Hz 1H, H-6bβ), 4.07–4.03 (m, 1H, H-5β), 2.19 (s, 3H, COCH_3_), 2.12 (s, 3H, COCH_3_), 2.10 (s, 3H, COCH_3_), 2.05 (s, 3H, COCH_3_), and 2.04 (s, 3H, COCH_3_); ^13^C NMR (100 MHz, CDCl_3_): δ 170.6 (COCH_3_), 170.0 (COCH_3_), 169.6 (COCH_3_), 169.5 (COCH_3_), 169.3 (COCH_3_), 166.2 (d, *J* = 153 Hz, C-F), 163.0 (S-C=N), 145.6 (N-C=N), 132.8 (Ph), 132.7 (Ph), 129.0 (Ph), 128.2 (Ph), 116.3 (Ph), 116.1 (Ph), 115.8 (Ph), 115.5 (Ph), 84.2 (C-1), 83.5 (C-1), 76.1 (C-3), 74.3 (C-3), 73.2 (C-5), 70.6 (C-5), 68.7 (C-4), 67.3 (C-4), 61.8 (C-6), 61.5 (C-6), 25.9 (C-2), 25.4 (C-2), 21.4 (COCH_3_), 21.0 (COCH_3_), 20.8 (COCH_3_), 20.7 (COCH_3_), and 20.6 (COCH_3_); HRMS *m/z* calcd for (C_20_H_21_FIN_3_O_7_S + H^+^): 594.0202; found: 509.0209.

*5-(4-chlorophenyl)-N-(3,4,6-tri-O-acetyl-2-deoxy-2-iodo-β-L-glucopyranosyl)-1,3,4-thiadiazol-2-amine* (**3h**): Starting from 3,4,6-tri-*O*-acetyl-D-glucal (**1b**: 288 mg, 1.058 mmol) and 2-amino-5-(4-chlorophenyl)-1,3,4-thiadiazole (**2d**: 448 mg, 2.116 mmol), yield 69% (154.6 mg); ratio of anomers (α:β = 1:99); α^24^_D_ = 177.7 (c = 0.6, CHCl_3_); ^1^H NMR (400 MHz, CDCl_3_): δ 7.59–7.56 (m, 2H, Ph), 7.43–7.40 (m, 2H, Ph), 5.94 (d, *J* = 10.0 Hz, 1H, H-1β), 5.47 (dd, *J* = 8.8 Hz, 10.8 Hz, 1H, H-3β), 5.13 (dd, *J* = 9.2 Hz, 10 Hz, 1H, H-4β), 4.68–4.62 (m, 1H, H-2β), 4.35 (dd, *J* = 4.4 Hz, 12.4 Hz, 1H, H-6aβ), 4.14 (dd, *J* = 2.4 Hz, 12.8 Hz 1H, H-6bβ), 4.01–3.97 (m, 1H, H-5β), 2.12 (s, 3H, COCH_3_), 2.07 (s, 3H, COCH_3_), and 2.04 (s, 3H, COCH_3_); ^13^C NMR (150 MHz, CDCl_3_): δ 170.6 (COCH_3_),170.5 (COCH_3_), 169.8 (COCH_3_), 169.7 (COCH_3_), 169.5 (COCH_3_), 156.6 (S-C=N), 146.5 (N-C=N), 139.3 (C-Cl), 129.8 (Ph), 129.6 (Ph), 129.1 (Ph), 128.3 (Ph), 127.9 (Ph), 98.7 (C-1), 80.9 (C-1), 78.2 (C-3), 73.5 (C-3), 68.2 (C-5), 67.5 (C-5), 64.3 (C-4), 62.5 (C-6), 60.5 (C-6), 29.8 (C-2), 26.7 (C-2), 21.5 (COCH_3_), 21.3 (COCH_3_), 20.9 (COCH_3_), 20.7 (COCH_3_), and 20.6 (COCH_3_); HRMS *m/z* calcd for (C_20_H_21_ClIN_3_O_7_S + H^+^): 609.9906; found: 609.9917.

*5-(4-(tert-buthyl)phenyl)-N-(3,4,6-tri-O-acetyl-2-deoxy-2-iodo-α/β-D-galactopyranosyl)-1,3,4-thiadiazol-2-amine* (**3i**): Starting from 3,4,6-tri-*O*-acetyl-D-galactal (**1c**: 163 mg, 0.599 mmol) and 2-amino-5-(4-tert-buthylphenyl)-1,3,4-thiadiazole (**2a**: 279 mg, 1.196 mmol), yield 72% (100.7 mg); ratio of anomers (α:β = 2.3:1); ^1^H NMR (600 MHz, CDCl_3_): δ 7.80–7.62 (m, 2H, Ph), 7.50–7.46 (m, 2H, Ph), 6.72 (d, 1H, *J* = 7.8 Hz, H-1α), 6.55 (dd, 1H, *J* = 1.2 Hz, 6.0 Hz, H-1β), 5.80 (t, 1H, *J* = 3.0 Hz, H-4β), 5.55–5.53 (m, 1H, H-4α), 5.49 (dd, 1H, *J* = 3.6 Hz, 5.4 Hz, H-3α), 5.42–5.41 (m, 1H, H-3β), 5.09 (dd, 1H, *J* = 3.6 Hz, 8.4 Hz, H-2α), 4.72–4.71 (m, 1H, H-2β), 4.54–4.51 (m, 1H, H-5α), 4.49–4.47 (m, 1H, H-5β), 4.32–4.30 (m, 1H, H-6aα), 4.27–4.19 (m, 3H, H-6bα, H-6aβ, H-6bβ), 2.33 (s, 6H, COCH_3_α, COCH_3_β), 2.26 (s, 6H, COCH_3_α, COCH_3_β), 2.12 (s, 6H, COCH_3_α, COCH_3_β), 2.07 (s, 3H, COCH_3_α), 2.02 (s, 3H, COCH_3_β), 1.34 (s, 9H, C(CH_3_)_3_), and 1.33 (s, 9H, C(CH_3_)_3_); ^13^C NMR (150 MHz, CDCl_3_): δ 170.6 (COCH_3_), 170.3 (COCH_3_), 170.2 (COCH_3_), 169.5 (COCH_3_), 165.7 (S-C=N), 156.4 (C-C(CH_3_)_3_), 155.2 (C-C(CH_3_)_3_), 145.5 (S-C=N), 129.1 (Ph), 128.3 (Ph), 126.7 (Ph), 126.3 (Ph), 98.9 (C-1), 81.5 (C-1), 73.0 (C-3), 72.9 (C-3), 69.4 (C-5), 66.2 (C-5), 64.0 (C-4), 63.9 (C-4), 62.0 (C-6), 60.3 (C-6), 35.1 (C-2), 31.2 (C(CH_3_)_3_), 29.8 (C(CH_3_)_3_), 27.1 (C-2), 21.7 (COCH_3_), 21.1 (COCH_3_), 20.9 (COCH_3_), 20.8 (COCH_3_), and 20.7 (COCH_3_); HRMS *m/z* calcd for (C_24_H_30_IN_3_O_7_S + H^+^): 632.0922; found: 632.0928.

*5-phenyl-N-(3,4,6-tri-O-acetyl-2-deoxy-2-iodo-α-D-galactopyranosyl)-1,3,4-thiadiazol-2-amine* (**3j**): Starting from 3,4,6-tri-*O*-acetyl-D-galactal (**1c**: 264 mg, 0.970 mmol) and 2-amino-5-phenyl-1,3,4-thiadiazole (**2b**: 344 mg, 1.941 mmol), yield 62% (106.5 mg); ratio of anomers (α:β = 3.8:1); ^1^H NMR (400 MHz, CDCl_3_): δ 7.65–7.63 (m, 2H, Ph) 7.46–7.42 (m, 3H, Ph), 6.23 (d, 1H, *J* = 8.4 Hz, H-1α), 5.94 (d, 1H, *J* = 10.8 Hz, H-1β), 5.76 (t, 1H, *J* = 3.2 Hz, H-4α), 5.45 (dd, 1H, *J* = 3.2 Hz, 5.6Hz, H-3α), 5.34–5.28 (m, 2H, H-4β, H-3β), 5.01–4.98 (m, 1H, H-2α), 4.73–4.68 (m, 1H, H-6aα), 4.52–4.47 (m, 1H, H-5α), 4.42–4.38 (m, 1H, H-6bα), 4.27–4.11 (m, 4H, H-2β, H-5β, H-6aβ, H-6bβ), 2.24 (s, 3H, COCH_3_α), 2.20 (s, 3H, COCH_3_β), 2.09 (s, 6H, COCH_3_α, COCH_3_β), 2.08 (s, 3H, COCH_3_α), and 2.04 (s, 3H, COCH_3_β); ^13^C NMR (100 MHz, CDCl_3_): δ 169.8 (COCH_3_), 169.6 (COCH_3_), 169.5 (COCH_3_), 162.7 (S-C=N), 147.2 (N-C=N), 131.0 (Ph), 129.5 (Ph), 127.5 (Ph), 83.2 (C-1), 73.7 (C-3), 71.3 (C-5), 68.1 (C-4), 62.6 (C-6), 24.9 (C-2), 21.2 (COCH_3_), 20.6 (COCH_3_), and 20.5 (COCH_3_); HRMS *m/z* calcd for (C_20_H_22_IN_3_O_7_S + H^+^): 576.0296; found: 576.0312.

*5-(4-fluorophenyl)-N-(3,4,6-tri-O-acetyl-2-deoxy-2-iodo-α/β-D-galactopyranosyl)-1,3,4-thiadiazol-2-amine* (**3k**): Starting from 3,4,6-tri-*O*-acetyl-D-galactal (**1c**: 213 mg, 0.782 mmol) and 2-amino-5-(4-fluorophenyl)-1,3,4-thiadiazole (**2c**: 305 mg, 1.562 mmol), yield 45% (68.7 mg); ratio of anomers (α:β = 4:1); ^1^H NMR (400 MHz, CDCl_3_): δ 7.67–7.6 (m, 2H, Ph), 7.37–7.36 (m, 2H, Ph), 6.21 (d, 1H, *J* = 8.8 Hz, H-1α), 5.94 (d, 1H, *J* = 10.4 Hz, H-1β), 5.75 (t, 1H, *J* = 3.2 Hz, H-4β), 5.44 (dd, 1H, *J* = 3.2 Hz, 5.6Hz, H-4α), 5.33–5.28 (m, 2H, H-3β, H-3α), 4.98 (dd, 1H, *J* = 3.2 Hz, 8.8Hz, H-2α), 4.81–4.75 (m, 2H, H-2β, H-6α), 4.51–4.46 (m, 1H, H-5α), 4.36 (dd, 1H, *J* = 3.2 Hz, 12.4Hz, 6bα), 4.27–4.12 (m, 3H, H-5β, H-6bβ, H-6abβ), 2.24 (s, 6H, COCH_3_α, COCH_3_β), 2.24 (s, 6H, COCH_3_α, COCH_3_β), 2.20 (s, 6H, COCH_3_α, COCH_3_β), 2.09 (s, 3H, COCH_3_α), and 2.03 (s, 3H, COCH_3_β); HRMS *m/z* calcd for (C_20_H_21_FIN_3_O_7_S + H^+^): 594.0202; found: 594.0217.

*5-(4-chlorophenyl)-N-(3,4,6-tri-O-acetyl-2-deoxy-2-iodo-α/β-D-galactopyranosyl)-1,3,4-thiadiazol-2-amine* (**3l**): Starting from 3,4,6-tri-*O*-acetyl-D-galactal (**1c**: 202 mg, 0.742 mmol) and 2-amino-5-(4-chlorophenyl)-1,3,4-thiadiazole (**2d**: 314 mg, 1.483 mmol), yield 58% (90.9 mg); ratio of anomers (α:β = 1.4:1); ^1^H NMR (600 MHz, CDCl_3_): δ 7.66–7.63 (m, 2H, Ph), 7.45–7.42 (m, 2H, Ph), 6.45 (d, 1H, *J* = 8.4 Hz, H-1α), 6.23 (d, 1H, *J* = 8.4 Hz, H-1β), 5.74 (t, 1H, *J* = 3.0 Hz, H-4α), 5.55–5.54 (m, 1H, *J* = 3.0 Hz, H-4β), 5.45 (dd, 1H, *J* = 3.0 Hz, 6.0 Hz, H-3α), 5.42–5.41 (m, 1H, H-3β), 5.00 (dd, 1H, *J* = 3.0 Hz, 8.4Hz, H-2α), 4.73–4.71 (m, 1H, H-2β), 4.50–4.47 (m, 1H, H-5α), 4.31 (t, 1H, *J* = 6.0 Hz, H-6aα), 4.28–4.19 (m, 4H, H-5β, H-6bα, H-6aβ, H-6bβ), 2.26 (s, 3H, COCH_3_β), 2.12 (s, 6H, COCH_3_α, COCH_3_β), 2.08 (s, 6H, COCH_3_α, COCH_3_β), and 2.02 (s, 3H, COCH_3_α); ^13^C NMR (150 MHz, CDCl_3_): δ 170.6 (COCH_3_),170.4 (COCH_3_), 170.2 (COCH_3_), 169.4 (COCH_3_), 169.3 (COCH_3_), 165.6 (S-C=N), 145.5 (N-C=N), 138.1 (C-Cl), 129.7 (Ph), 129.5 (Ph), 129.1 (Ph), 128.0 (Ph), 127.6 (Ph), 99.0 (C-1), 80.5 (C-1), 74.1 (C-3), 72.9 (C-3), 69.7 (C-5), 66.2 (C-5), 64.0 (C-4), 62.0 (C-6), 60.2 (C-6), 29.8 (C-2), 25.7 (C-2), 21.2 (COCH_3_), 21.1 (COCH_3_), 20.9 (COCH_3_), 20.8 (COCH_3_), and 20.7 (COCH_3_); HRMS *m/z* calcd for (C_20_H_21_ClIN_3_O_7_S + H^+^): 609.9906; found: 609.9917.

*5-phenyl-N-(3,4-di-O-benzyl-2-deoxy-2-iodo-α/β-L-rhamnopyranosyl)-1,3,4-thiadiazol-2-amine* (**3m**): Starting from 3,4-di-*O*-benzyl-L-rhamnal (**1d**: 131 mg, 0.442 mmol) and 2-amino-5-(4-tert-buthylphenyl)-1,3,4-thiadiazole (**2a**: 197 mg, 0.844 mmol), yield 42% yield (41.5 mg); ratio of anomers (α:β = 1:1); ^1^H NMR (400 MHz, CDCl_3_): δ 7.70–7.68 (m, 2H, Ph), 7.47–7.42 (m, 6H, Ph), 7.37–7.31 (m, 20H, Ph), 5.99 (d, 1H, *J* = 10.2 Hz, H-1), 5.08 (dd, 2H, *J* = 2.4 Hz, 10.2 Hz, -CH_2_-Ph), 4.93–4.88 (m, 4H, -CH_2_-Ph), 4.72–4.66 (m, 3H, H-2, -CH_2_-Ph), 4.62 (d, 1H, J = 9.6 Hz, H-1β), 4.21 (t, 1H, *J* = 10.2 Hz, H-2β), 3.90 (t, 1H, *J* = 9.6 Hz, H-3), 3.82 (dd, 1H, *J* = 9.0 Hz, 10.8 Hz, H-3), 3.72 (m, 1H, H-5), 3.62 (dq, 1H, *J* = 6.6/6.0 Hz, 9.6 Hz, H-5), 3.33 (dt, 2H, *J* = 9.0 Hz, 14.4 Hz, H-4), 1.37 (d, 3H, *J* = 6.6 Hz, CH_3_), and 1.33 (d, 3H, *J* = 6 Hz, CH_3_); ^13^C NMR (100 MHz, CDCl_3_): δ 160.8 (S-C=N),148.5 (N-C=N), 138.1 (Ph), 138.0 (Ph), 130.7 (Ph), 130.5 (Ph), 129.0 (Ph), 128.7 (Ph), 128.6 (Ph), 128.5 (Ph), 128.3 (Ph), 128.1 (Ph), 126.5 (Ph), 95.5 (C-1), 94.9 (C-1), 86.4 (C-3), 85.1 (C-3), 85.0 (C-4), 75.5 (C-5), 74.3 (-CH_2_-), 73.9 (-CH_2_-), 37.6 (C-2), 31.1 (C-2), and 17.9 (C-6); HRMS *m/z* calcd for (C_28_H_28_IN_3_O_3_S + H^+^): 614.0969; found: 614.0969.

### 3.2. Biological Evaluation

#### 3.2.1. Cell Lines and Culture Conditions

Cytotoxicity of drugs was determined for several cell lines. Experiments were performed on HeLa (cervical cancer cell line), HCT 116 (colorectal carcinoma cell line), and MCF-7 (human breast adenocarcinoma cell line) according to the standard procedures described previously [62]. The cells were obtained from American Type Culture Collection (ATCC, Manassas, VA, USA). The culture was performed in Dulbecco’s Modified Eagle Medium, Nutrient Mixture F-12 (DMEM/F12, Sigma-Aldrich, Taufkirchen, Germany) supplemented with fetal bovine serum (FBS, Gibco) as a source of growth factors and gentamicin (40 mg/mL, Krka Poland Sp. z o.o., Warsaw, Poland) in standard quantities. The culture medium for all cell lines contained 12% of heat-inactivated FBS. Monolayer cell cultures have grown in 75 cm^2^ surface bottles (Nunc, ThermoFisher, Waltham, MA, USA), in an incubator (Heracell 150i ™, Thermo Scientific ™, Merck, Darmstadt, Germany) of a solid concentration of 5% carbon dioxide, 37 °C, and constant humidity of 80%. The culture was maintained in vitro by the passaging of cells (every 3 to 4 days). For the experiments, cells were obtained by trypsinization with 0.25% trypsin solution (Trypsin EDTA, Immuniq, Żory, Poland) in phosphate-buffered saline lacking Ca^+2^ and Mg^+2^ (PBS) to detach them from the ground. Trypsin was neutralized by adding an equal amount of the culture medium. After centrifugation (1500 rpm, 5 min.), the cells were resuspended in a fresh culture medium and counted using an automatic cell counter—Juli Stage (NanoEntek, Waltham, MA, USA).

#### 3.2.2. Cytotoxicity Test by MTT Assay

For the cytotoxicity determination of the tested derivatives, the cells in the exponential growth phase were trypsinized and seeded on 96-well plates (Nunc, ThermoFisher, Waltham, MA, USA) at concentration 5 × 10^4^ cells/well/200 μL in completed grown medium. After approx. 24 h the culture medium was replaced with a fresh culture medium containing serial dilutions of the tested drugs. After 72 h, a solution of the compound was removed. Next, cell viability was determined by the MTT assay based on mitochondrial dehydrogenase enzyme activity. The basis of the method is the reaction of the reduction of tetrazolium salts to colored formazan carried out by dehydrogenase in living cells. Therefore, the amount of formazan formed is proportional to the number of functional cells. MTT assay was carried out according to the protocol (MTT, Sigma-Adrich, Taufkirchen, Germany) [63,64]. To each well on the plate, 50 µL of MTT solution (0.05 mg/mL in phenol red and FBS free DMEM-F12; PAA) was added. The plates were then placed in a CO_2_ incubator for 1 to 2 h. After this time, MTT solution was removed, and formazan crystals were dissolved in acidic isopropanol. Next, spectrophotometrically absorbance measuring at 570 nm, using a multi-well plate reader SYNERGY4 (BioTek Instruments, Winooski, VT, USA) was performed. The fraction of viable cells was determined with concern to not treat with any factor control cells, according to the following formula:

(1) $$\mathrm{SF}=\frac{A}{A_{K}^{}}\times 100\%$$

SF—survival fraction;

A—the absorbance value;

A_K_—the absorbance value for control cells.

#### 3.2.3. Cell Cycle and Apoptosis Analysis

The exponentially growing cells were harvested by trypsinization and seeded on a 6-well plate (Nunc, ThermoFisher, Waltham, MA, USA) in DMEM/F12 medium at concentrations of 1 × 10^5^ cells/well/2 mL. After 72 h, the growth medium was exchanged for a fresh medium in control groups or medium containing the tested drugs at chosen concentrations. The cells were incubated for 72 h. After this time, the medium was collected and the cells were trypsinized with 1 mL trypsin/EDTA (Sigma-Aldrich, Taufkirchen, Germany). Trypsin was then neutralized by adding 2 mL of culture medium. The cells were centrifuged at 1300 rpm for 5 min at 4 °C, washed twice with 1 mL PBS, and finally fixed with 1 mL of ice-cold 70% ethanol. The samples were stored at −20 °C until analysis. Directly before the analysis by flow cytometer (Becton Dickinson Aria III, BD Company, San Diego, CA, USA), cells were centrifuged at 1300 rpm for 3 min, washed with 500 µL PBS, and centrifuged again. Then, cells were resuspended in 50 µL PBS and 50 µL of RNAse solution (Sigma-Aldrich, Taufkirchen, Germany) at a concentration of 100 µg/mL (in PBS). After 15 min of incubation at 37 °C, cells were stained with 250 µL propidium iodide solution (PI, Sigma-Aldrich, Taufkirchen, Germany) at a concentration of 100 µg/mL. Fluorescence was measured using a flow cytometer with the PE configuration (547 nm excitation laser line; emission: 585 nm). The samples were vortexed before analysis [65,66].

For apoptosis assay, the cells after collection were stained directly using FITC conjugated primary Annexin-V antibody (BioLegend, San Diego, USA) in binding buffer for 20 min in darkness at 37 °C and Annexin with PI solution 100 µg/mL (Sigma-Aldrich, Taufkirchen, Germany). Fluorescence was measured using a flow cytometer with the PE configuration (547 nm excitation laser line; emission: 585 nm) for necrotic and late apoptotic cell counting. For apoptotic cells, the FITC channel configuration was used (488 nm excitation laser line; emission: LP mirror 503, BP filter 530/30). The samples were vortexed before analysis [65,66].

#### 3.2.4. Statistical Analysis

At least three replicates were performed for every kind of experiment. The results were presented as the mean value ± SD. The statistical analysis was based on a t-test, and a *p*-value less than 0.05 was considered statistically significant.

## Figures and Tables

**Table 1:** Study on the reaction of 3,4-di-*O*-acetyl-L-rhamnal iodoamination(**1a**).

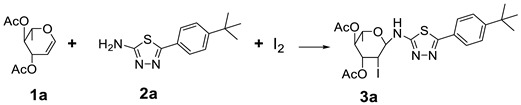			
**Entry**	**Solvent**	**Iodine Donor**	**Yield**
a	THF	-	n.r.
b	CH2Cl2/DMF	-	n.r.
c	CH2Cl2/DMSO	-	n.r.
d	CH2Cl2	1eq of I2	traces
e	CH2Cl2/DMF	1eq of I2	traces
f	CH2Cl2/DMSO	1eq of I2	traces
g	THF	0.5eq of I2	n.r.
h	THF	1eq of I2	35%(α/β=1:9)
i	THF	2eq of I2	71%(α/β = 1:11)
j	THF	2eq of NIS	30%(α/β=1:1)

**Table 2:** Iodoamination of selected glycals.

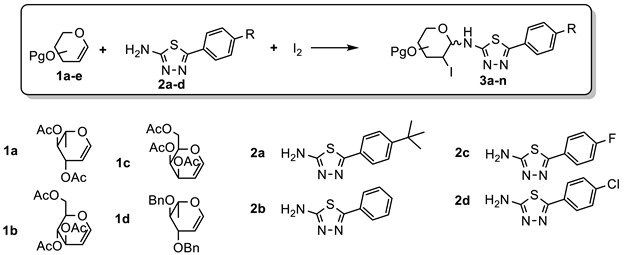					
**Entry**	**Glycal (1)**	**Nucleophile (2)**	**Product (3)**	**Yield (%)**	**Ratio (α/β)**
a	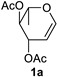	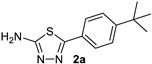	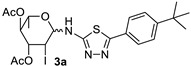	71	1/11
b	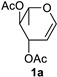	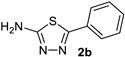	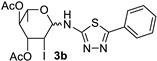	47	1/1.7
c	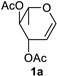	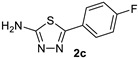	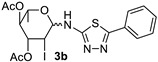	55	1/99
d	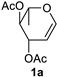	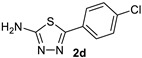	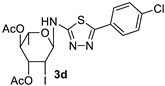	49	1/99
e	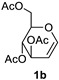	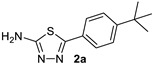	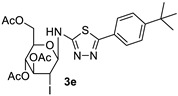	51	1/99
f	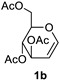	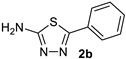	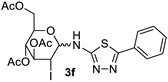	68	4.4/1
g	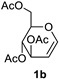	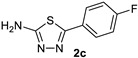	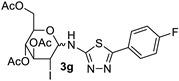	58	1/1.3
h	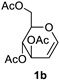	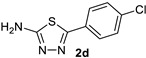	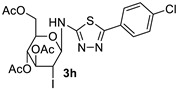	69	1/99
i	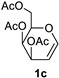	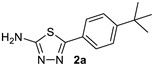	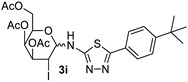	72	2.3/1
j	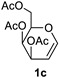	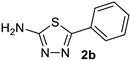	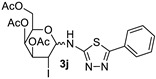	62	3.8/1
k	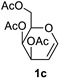	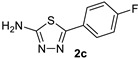	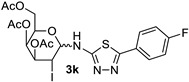	45	4/1
l	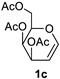	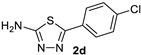	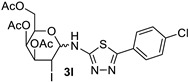	58	1.4/1
m	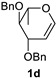	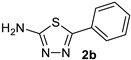	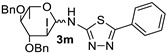	42	1/1

**Table 3:** The comparison of the cytotoxic effects of **3a**–**3c, 3f**–**3h, 3j, 3l and 3m** derivatives against MCF-7, HCT116, and HeLa cell lines.

Compound	MCF-7			HCT116			HeLa		
	MTT (Compound conc. 100 µM)	Cells in sub-G1 Phase	Early Apoptotic Cells	MTT (Compound conc. 100 µM)	Cells in sub-G1 Phase	Early Apoptotic Cells	MTT (Compound conc. 100 µM)	Cells in sub-G1 Phase	Early Apoptotic Cells
**3a**	(-)	↑↑	↓	↓	↑	↑↑	(-)	↑↑↑	↑↑↑
**3b**	(-)	↑↑	↓	↓	↑	↑↑	↓	↑↑↑	↑↑
**3c**	↓↓	↑↑	↑	↓↓	↑	(-)	↓↓	↑↑↑	↑↑
**3f**	(-)	↑	↓	↓	(-)	↑↑	↓	↑↑↑	↑↑
**3g**	(-)	↓	↓	↓	(-)	↑↑	↓	↑↑↑	↑↑
**3h**	(-)	↑	↓	(-)	↑	↓	↓	↑↑↑	↑↑
**3j**	↓↓	↑	↓	↓	↑↑	↑↑	↓	↑↑↑	↑↑
**3l**	↓	(-)	↓	↓	↑↑	↑↑	(-)	↑↑↑	↑↑
**3m**	(-)	(-)	↓	↓	↑	↑	↓	↑↑↑	↑↑

(-);(-);(-) **—**no effect. **↓—**decrease in SF value. The number of arrows indicates the strength of the effect (single weak, double stronger). **↑—**increase in the number of cells in sub-G1 phase compared to cells in the control group **↓—a** decrease in the number of cells in sub-G1 phase compared to cells in the control group. The number of arrows indicates the strength of the effect (single: weak, double: stronger, triple: the strongest). **↑—**increase in the number of early apoptotic cells compared to cells in the control group **↓—a** decrease in the number of early apoptotic cells compared to cells in the control group. The number of arrows indicates the strength of the effect (single: weak, double: stronger, triple: the strongest).

## Data Availability

Data is contained within the article or supplementary material.

## Suplementary Materials

The Supporting Information is available online. ^1^H and ^13^C NMR spectra of all obtained compounds, Figure S1: Typical histograms of PI stained DNA content during the cell cycle of MCF-7, HCT116, and HeLa cells, after 72 h of incubation with compounds at a dose of 100 µM; Figure S2:Typical dot plots of normal, early apoptotic, late apoptotic and necrotic MCF-7, HCT116, and HeLa cells after 72 h of incubation with compounds at a dose of 100 µM; General procedure for the synthesis of 2-benzoylhydrazinecarbothioamide derivatives (6).

## Sample Availability

Samples of the compounds **3a**–**3m** are available from the authors.
