# Supplementary material for: Synthesis and Preliminary Anticancer Activity Assessment of N-Glycosides of 2-Amino-1,3,4-thiadiazoles
Source: Molecules. 2021 Nov 29;26(23):7245. doi: 10.3390/molecules26237245 (PMC8659227; doi:10.3390/molecules26237245)
Supplement: Supplementary file 1 [file molecules-26-07245-s001.zip › molecules-1476010-supplementary.pdf]

Supplementary Data

Synthesis and preliminary anticancer activity assessment of N-glycosides of 2-amino-1,3,4-thiadiazoles

Katarzyna Żurawska[1,2], Marcin Stokowy[1], Patryk Kapica[1,2], Monika Olesiejuk[3], Agnieszka Kudelko[3], Katarzyna Papaj[2], Magdalena Skonieczna[2,4], Wiesław Szeja[5], Krzysztof Walczak[1] and Anna Kasprzycka[1,2*]

[1]Department of Organic Chemistry, Bioorganic Chemistry and Biotechnology, Faculty of Chemistry, Silesian University of Technology, Krzywoustego Street 4, 44-100 Gliwice, Poland.

[2]Centre of Biotechnology, Silesian University of Technology, Krzywoustego Street 8, 44-100 Gliwice, Poland

[3]Department of Chemical Organic Technology and Petrochemistry, The Silesian University of Technology, Krzywoustego Street 4, 44-100 Gliwice, Poland

[4]Department of Systems Biology and Engineering, The Silesian University of Technology, Akademicka Street 16, 44–100 Gliwice, Poland

[5]The Silesian University of Technology, Akademicka Street 16, 44–100 Gliwice, Poland.

*Correspondence: anna.kasprzycka@polsl.pl (A.K.)

Content:

**General procedure for the synthesis of 2-benzoylhydrazinecarbothioamide derivatives (6)**	**S2**
**NMR Spectra**	**S5**
**Figure S1.** Typical histograms of PI stained DNA content during the cell cycle of MCF-7, HCT116, and HeLa cells, after 72h of incubation with compounds at a dose of 100 μM.	**S16**
**Figure S2.** Typical dot plots of normal (Annexin-V negative and PI negative stained); early apoptotic (Annexin-V positive and PI negative stained); late apoptotic (Annexin-V positive and PI-positive stained); and necrotic (Annexin-V negative and PI-positive stained) MCF-7, HCT116, and HeLa cells after 72h of incubation with compounds at a dose of 100μM.	**S17**

General procedure for the synthesis of 2-benzoylhydrazinecarbothioamide derivatives (6)

A series of 2-amino-1,3,4-thiadiazole derivatives (**2**) were prepared from the commercially available aromatic carboxylic acids (**4**) and thionyl chloride followed by transformation into appropriate acyl thiosemicarbazide **6** in a two-phase solvent system (H_2O:toluene). Finally cyclization of the intermediate **6** in the concentrated H_2SO_4 solution at room temperature was carried out.

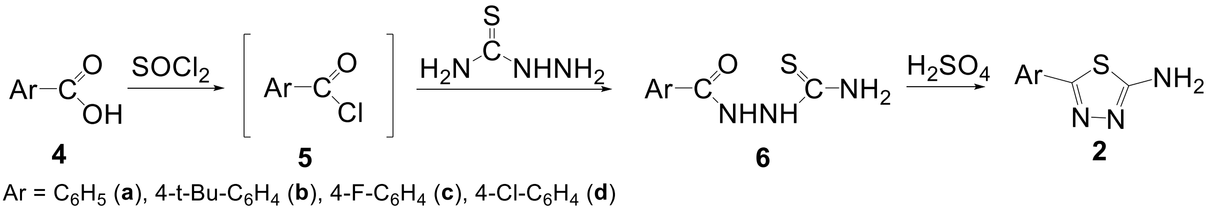

Scheme 5. Two-step synthesis of 2-amino-1,3,4-thiadiazole derivatives (**2a-d**).

The appropriate arylcarboxylic acid **4** (0.10 mol) and thionyl chloride $SOCl_2$ (22 mL, 0.30 mol) were refluxed in an anhydrous toluene (10 mL) until the acid was fully consumed (TLC; 5 - 15h). After cooling, the mixture was concentrated on a rotary evaporator, washed with an additional portion of anhydrous toluene (10 mL) and concentrated again. Then the crude acyl chloride **5** was dissolved in anhydrous toluene (50 mL) and added dropwise to the mixture of thiosemicarbazide (9.11 g, 0.10 mol), $NaHCO_3$ (8.40 g, 0.10 mol) and H_2O (150 mL) while stirring. The reaction mixture was stirred for 24 hours. The precipitated solid was filtered off, dried on air and recrystallized from the mixture of EtOH:H_2O to give pure 2-benzoylhydrazinecarbothioamide derivatives (**6**).

*2-(4-t-Butylbenzoylhydrazinecarbothioamide (**6a**)*

The product was obtained as a beige solid (48% yield, 12.01 g, 0.048 mol); mp 210-211 °C; R_f (MeOH:$CHCl_3$, 1:4 v/v) 0.56. ^1H NMR (400 MHz, DMSO-d_6): δ 10.39 (s, 1H, NH), 9.31 (s, 1H, NH), 7.84 (d, 2H, *J*=6.6 Hz, Ar: H-2, H-6), 7.52-7.60 (m, 2H, NH_2), 7.48 (d, 2H, *J*=6.6 Hz, Ar: H-3, H-5), 1.33 (s, 9H, $C(CH_3)_3$); ^{13}C NMR (100 MHz, DMSO-d_6): δ 182.0 (C=S), 165.6 (C=O), 154.6, 128.3, 126.1, 124.9, 34.8, 30.8; IR (ATR) ν: 3269, 3137, 2961, 1682, 1630, 1567, 1525, 1463, 1298, 1266, 1113, 1086, 1015 cm^{-1}; HRMS *m/z* calcd for ($C_{12}H_{17}N_3O_S$ + H^+): 252.1165; found: 252.1169.

*2-Benzoylhydrazinecarbothioamide (**6b**)*

The product was obtained as a white solid (44% yield, 5.95 g, 0.030 mol); mp 197-198°C (lit.:196-198 °C [25]); R_f (MeOH:$CHCl_3$, 1:4 v/v) 0.54. ^1H NMR (400 MHz, DMSO-d_6): δ 10.37 (s, 1H, NH), 9.32 (s, 1H, NH), 7.90 (d, 2H, *J*=7.2 Hz, Ar: H-2, H-6), 7.90 (d, 2H, *J*=7.2 Hz, Ar: H-2, H-6), 7.85 (br s, 1H, NH), 7.61 (br

s, 1H, NH), 7.56 (t, 1H, *J*=7.2 Hz, Ar: H-4), 7.45-7.49 (t, 2H, *J*=7.2 Hz, Ar: H-3, H-5); ^{13}C NMR (100 MHz, DMSO-d_6): δ 182.0 (C=S), 165.8 (C=O), 132.5, 131.7, 128.1, 127.8; IR (ATR) ν: 3404, 3254, 3143, 2960, 1686, 1644, 1598, 1579, 1540, 1472, 1262, 1181, 1068, 1000 cm^{-1}

*2-(4-Fluorobenzoyl)hydrazinecarbothioamide (**6c**)*

The product was obtained as a white solid (69% yield, 14.71 g, 0.066 mol); mp 169-171°C (lit.: 172 °C [26]); R_f (MeOH:CHCl$_3$, 1:4 v/v) 0.50. ^{1}H NMR (400 MHz, DMSO-d_6): δ 10.71 (s, 1H, NH), 9.43 (s, 1H, NH), 8.12 (d, 2H, *J*=8.4 Hz, Ar: H-2, H-6), 7.94 (br s, 1H, NH), 7.78 (br s, 1H, NH), 7.54 (d, 2H, *J*=8.4 Hz, Ar: H-3, H-5); ^{13}C NMR (100 MHz, DMSO-d_6): δ 182.0 (C=S), 164.4 (C=O), 164.1, 129.4, 128.1, 116.2; IR (ATR) ν: 3503, 3423, 3134, 2977, 1678, 1629, 1601, 1514, 1346, 1257, 1169, 1080, 1037 cm^{-1}.

*2-(4-Chlorobenzoyl)hydrazinecarbothioamide (**6d**)*

The product was obtained as a white solid (80% yield, 18.38 g, 0.080 mol); mp 217-219 °C (lit.: 218-220 °C [26]); R_f (MeOH:CHCl$_3$, 1:4 v/v) 0.52. ^{1}H NMR (400 MHz, DMSO-d_6): δ 10.45 (s, 1H, NH), 9.34 (s, 1H, NH), 7.82-7.88 (m, 3H, Ar: H-2, H-6, NH), 7.68-7.72 (m, 3H, Ar: H-3, H-5, NH); ^{13}C NMR (100 MHz, DMSO-d_6): δ 182.0 (C=S), 164.9 (C=O), 137.1, 131.1, 129.9, 129.5; IR (ATR) ν 3649, 3434, 3163, 1664, 1605, 1591, 1460, 1241, 1073, 1008 cm^{-1}.

General procedure for the synthesis of 2-amino-1,3,4-thiadiazole derivatives (**2**)

2-Benzoylhydrazinecarbothioamide **6** (0.05 mol) was dissolved in concentrated H_2SO_4 (50 mL) and agitated at room temperature till next day. Then the mixture was alkalized with 25% ammonia and the precipitated product was filtered off, dried on air and recrystallized from the mixture of EtOH:H_2O yielding the corresponding 2-amino-1,3,4-thiadiazole **2**.

*2-Amino-5-(4-*tert*-buthylphenyl)-1,3,4-thiadiazole (**2a**)*

The product was obtained as a white solid (41% yield, 4.78 g, 0.020 mol); mp 251-253 °C; R_f (MeOH:CHCl$_3$, 1:4 v/v) 0.54. ^{1}H NMR (400 MHz, DMSO-d_6): δ 7.67 (d, 2H, *J*=8.4 Hz, Ar: H-2, H-6), 7.48 (d, 2H, *J*=8.4 Hz, Ar: H-3, H-5), 7.35 (s, 2H, NH$_2$), 1.29 (s, 9H, C(CH$_3$)$_3$); ^{13}C NMR (100 MHz, DMSO-d_6): δ 168.1, 156.3, 152.2, 128.3, 126.0, 125.8, 34.5, 30.8; IR (ATR) ν: 3269, 3095, 2959, 1632, 1566, 1511, 1472, 1363, 1266, 1135, 1110, 1056, 985, 832, 730 cm^{-1}; HRMS *m/z* calcd for ($C_{12}H_{15}N_3S + H^+$): 234.1059; found: 234.1063.

*2-Amino-5-phenyl-1,3,4-thiadiazole (**2b**)*

The product was obtained as a white solid (45% yield, 3.99 g, 0.023 mol); mp 232-233 °C (lit.:230 °C [27]); R_f (MeOH:$CHCl_3$, 1:4 v/v) 0.56. ^{1}H NMR (400 MHz, DMSO-d_6): δ 7.61 (d, 2H, *J*=8.0 Hz, Ar: H-2, H-6), 7.41-7.75 (m, 5H, Ar: H-3, H-4, H-5, NH_2); ^{13}C NMR (100MHz, DMSO-d_6): δ 168.5, 156.3, 130.9, 129.5, 129.1, 126.3; IR (ATR) ν 3253, 3062, 2954, 1630, 1510, 1467, 1336, 1264, 1131, 1058, 1002, 980, 759 cm^{-1}.

*2-Amino-5-(4-fluorophenyl)-1,3,4-thiadiazole (**2c**)*

The product was obtained as a white solid (61% yield, 5.95 g, 0.030 mol); mp 229-231°C (lit.: 229-231 °C [28]); R_f (MeOH:$CHCl_3$, 1:4 v/v) 0.58. ^{1}H NMR (400 MHz, DMSO-d_6): δ 7.67 (d, 2H, *J*=8.4 Hz, Ar: H-2, H-6), 7.38 (s, 2H, NH_2), 7.30 (d, 2H, *J*=8.4 Hz, Ar: H-3, H-5); ^{13}C NMR (100 MHz, DMSO-d_6): δ 169.9, 164.2, 156.1, 129.3, 128.1, 116.2; IR (ATR) ν 3388, 3279, 3112, 1673, 1628, 1593, 1497, 1336, 1311, 1270, 1220, 1105, 1060, 840, 773 cm^{-1}.

*2-Amino-5-(4-chlorophenyl)-1,3,4-thiadiazole (**2d**)*

The product was obtained as a white solid (65% yield, 6.88 g, 0.033 mol); mp 227-229 °C (lit.: 227 °C [29]); R_f (MeOH:$CHCl_3$, 1:4 v/v) 0.60. ^{1}H NMR (400 MHz, DMSO-d_6): δ 7.71 (d, 2H, *J*=8.0 Hz, Ar: H-2, H-6); 7.58 (d, 2H, *J*=8.0 Hz, Ar: H-3, H-5), 7.48 (br s, 2H, NH_2); ^{13}C NMR (100 MHz, DMSO-d_6): δ 168.8, 155.1, 136.1, 132.0, 129.5, 128.7; IR (ATR) ν 3249, 3068, 2962, 1635, 1590, 1507, 1463, 1320, 1262, 1136, 1069, 1014, 980, 824, 772 cm^{-1}.

NMR Spectra

3a

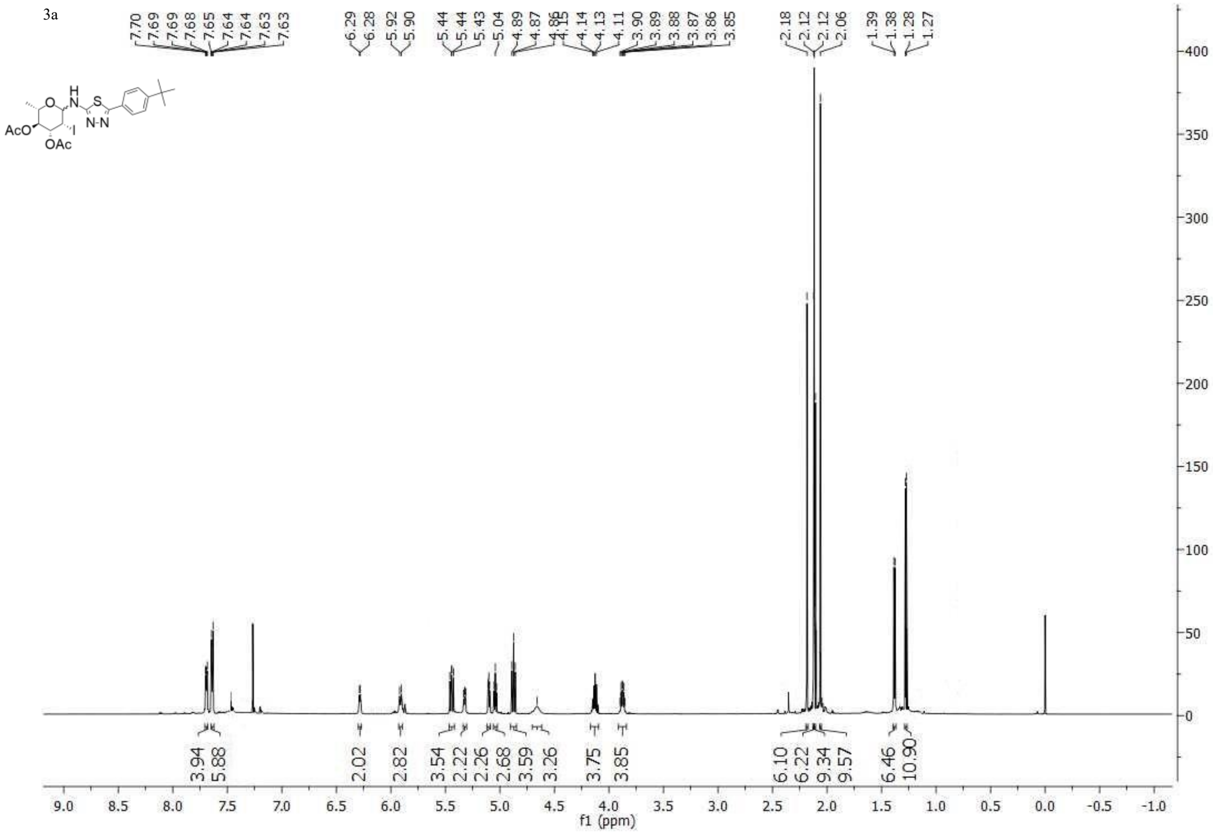

3a

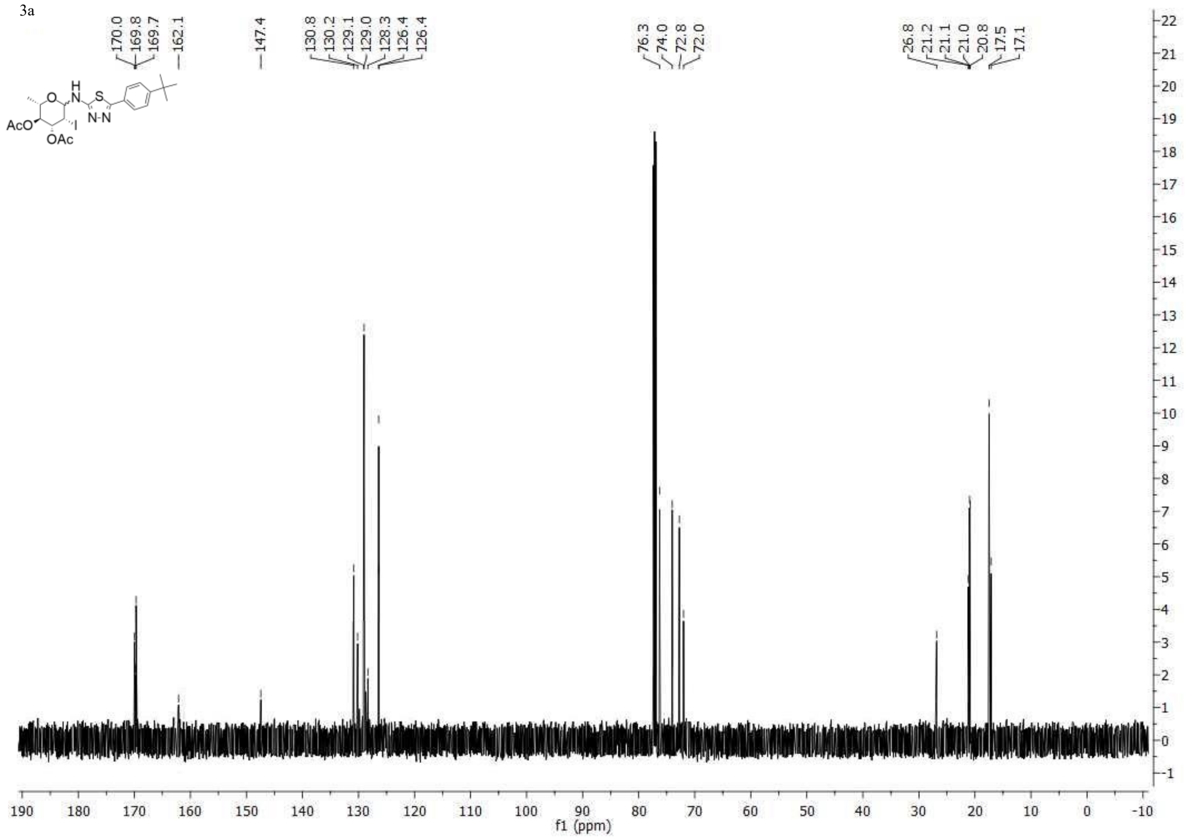

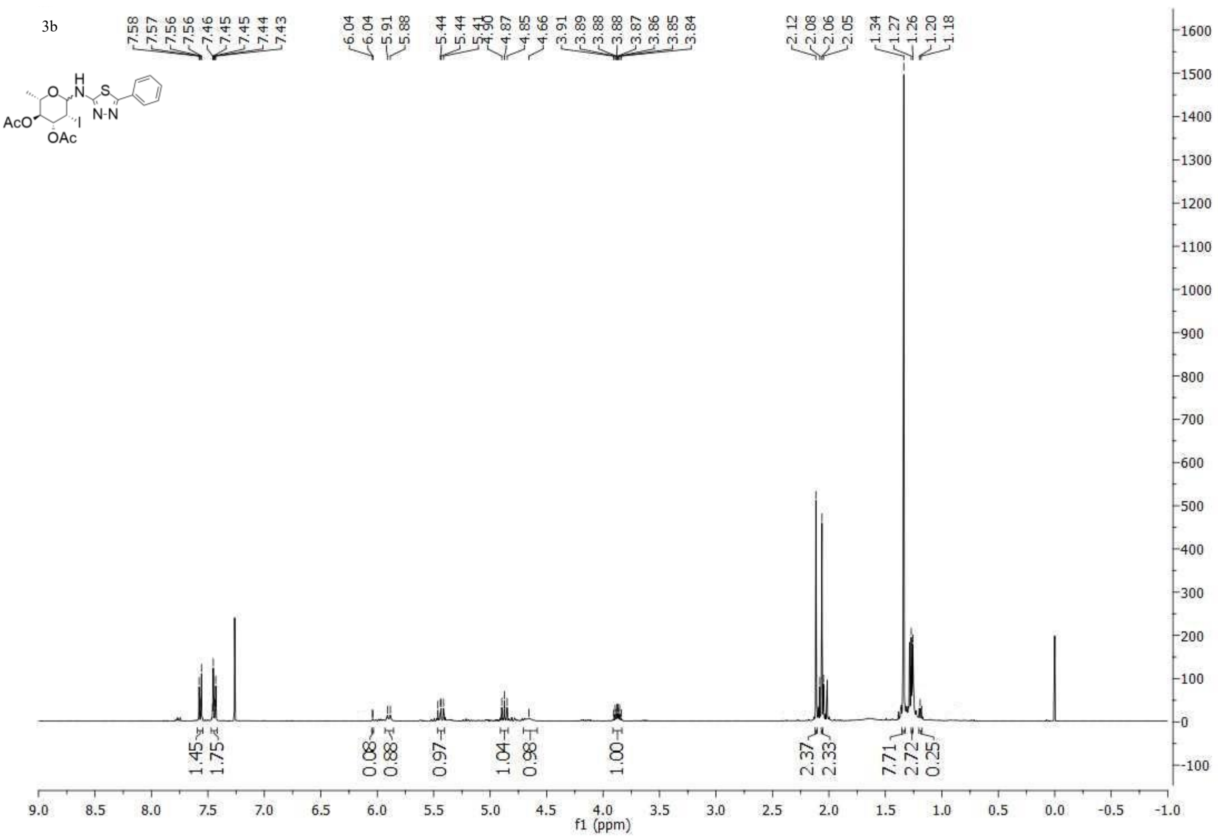
3b
7.58 7.57 7.56 7.56 7.46 7.45 7.45 7.44 7.43
6.04 6.04 5.91 5.88
5.44 5.44 5.41 4.90 4.87 4.85 4.66
3.91 3.89 3.88 3.88 3.87 3.86 3.85 3.84
2.12 2.08 2.06 2.05
1.34 1.27 1.26 1.20 1.18
1.45 1.75 0.08 0.88 0.97 1.04 0.98 1.00 2.37 2.33 7.71 2.72 0.25
f1 (ppm)

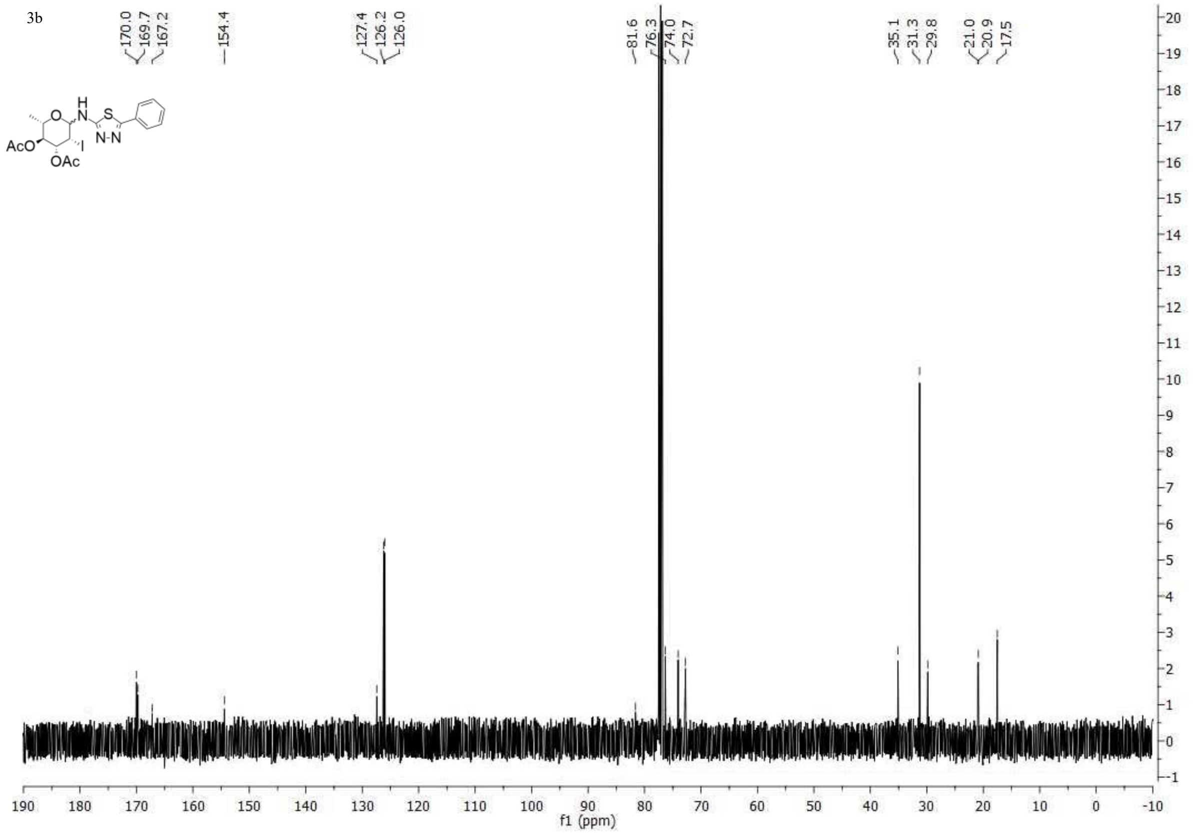
3b
170.0 169.7 167.2 154.4
127.4 126.2 126.0
81.6 76.3 74.0 72.7
35.1 31.3 29.8
21.0 20.9 17.5
f1 (ppm)

3c

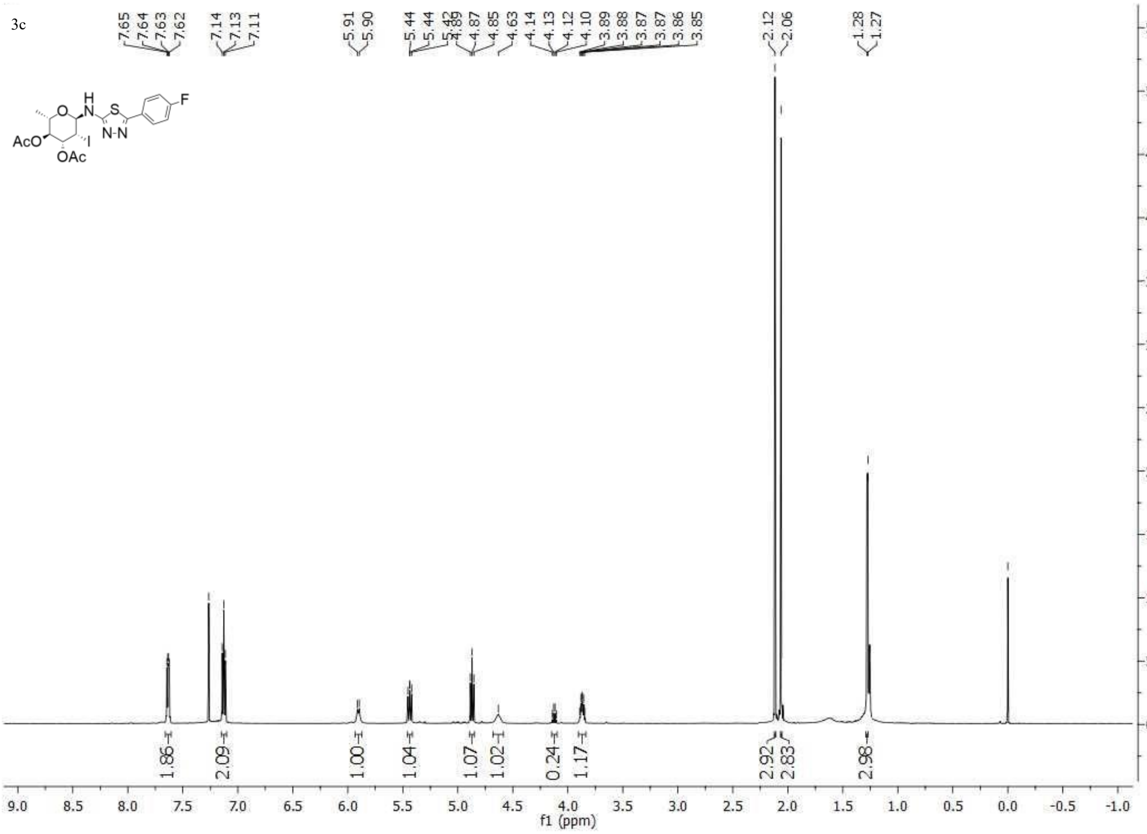

3c

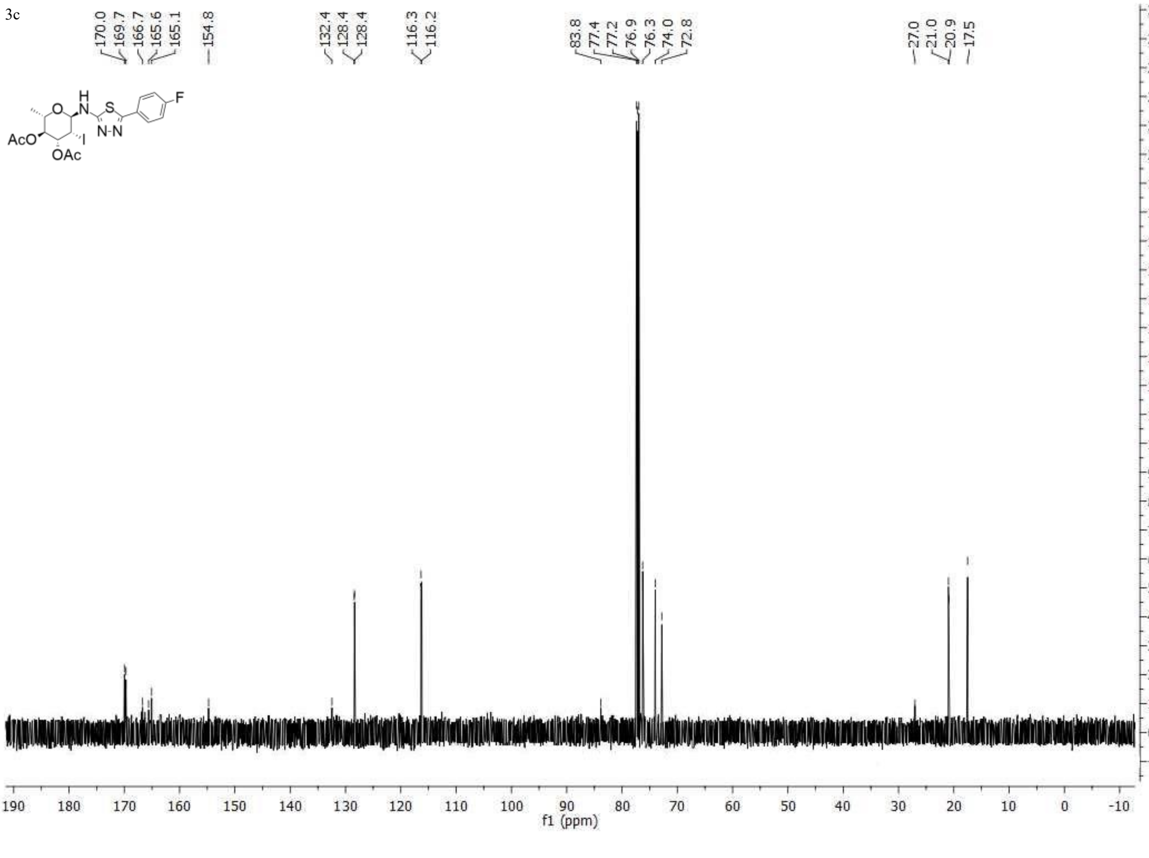

3d

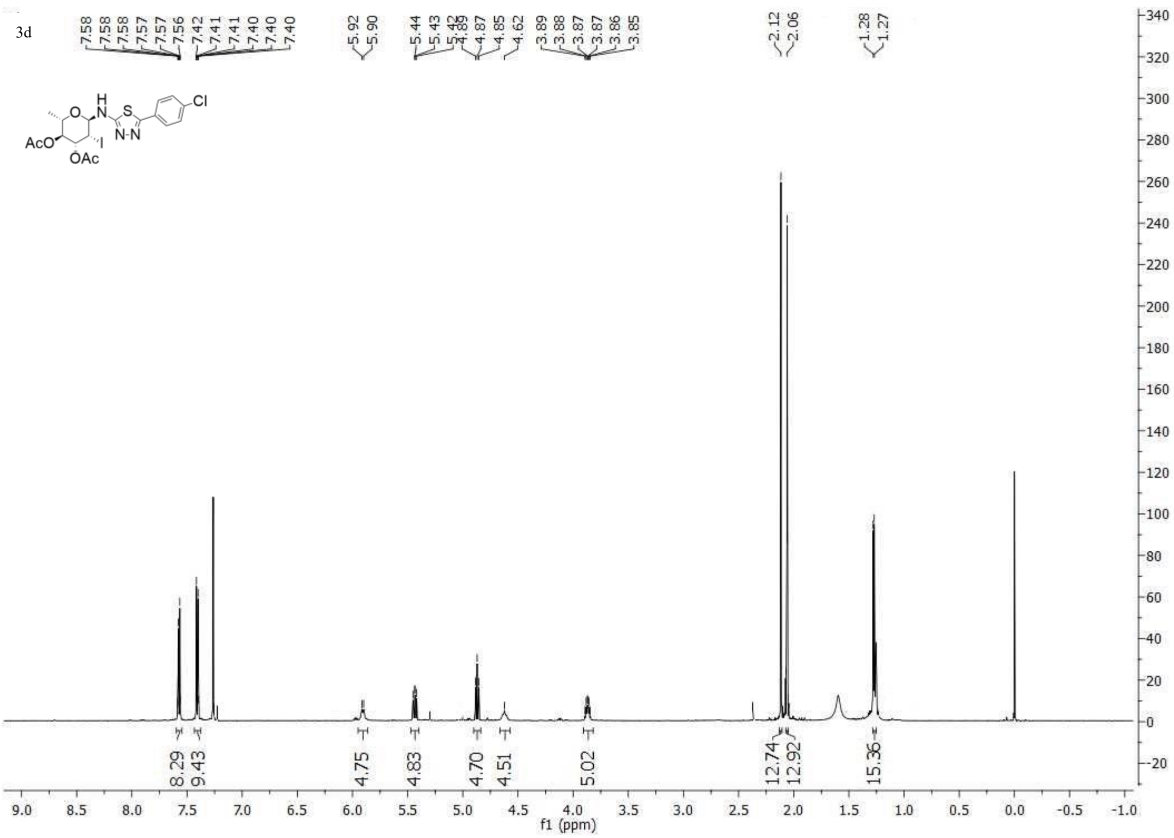
7.58 7.58 7.58 7.57 7.57 7.56 7.42 7.41 7.41 7.40 7.40 7.40
5.92 5.90
5.44 5.43 5.42 4.89 4.87 4.85 4.62
3.89 3.88 3.87 3.87 3.86 3.85
2.12 2.06
1.28 1.27
AcO
OAc
Cl
8.29 9.43 4.75 4.83 4.70 4.51 5.02 12.74 12.92 15.36
f1 (ppm)

3d

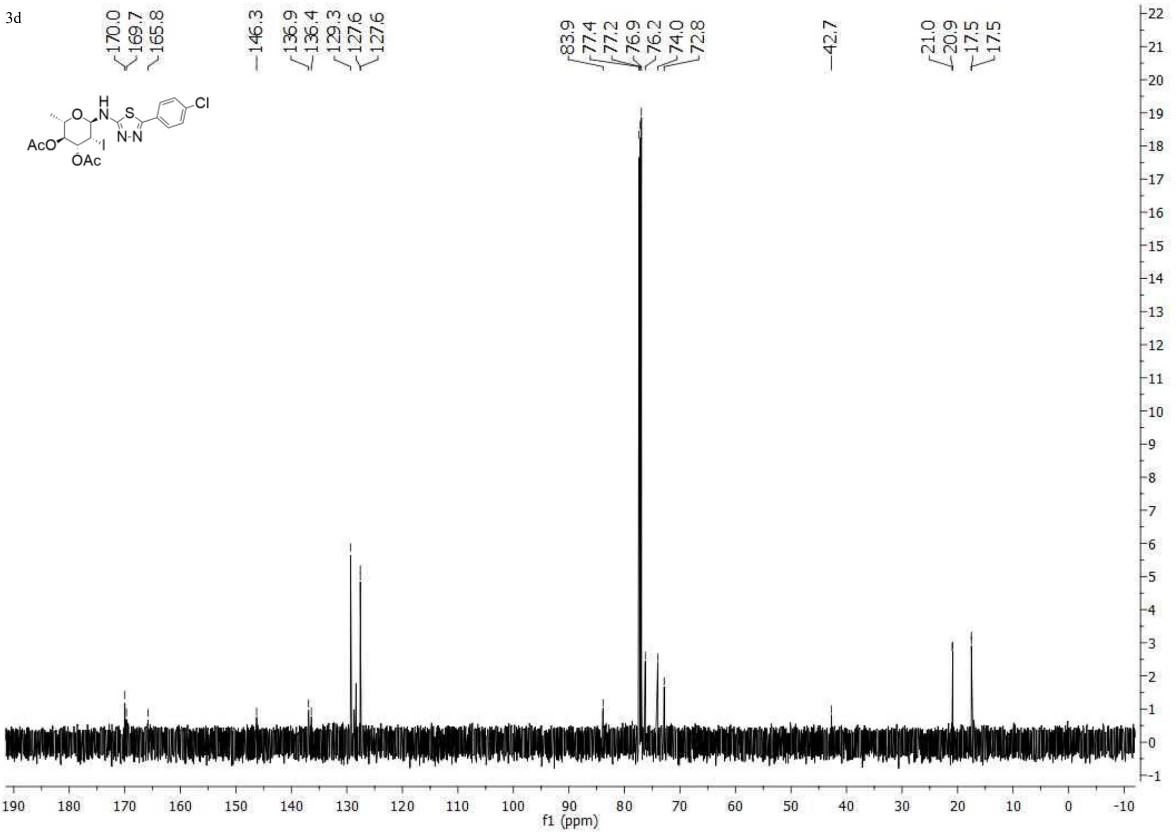
170.0 169.7 165.8
146.3
136.9 136.4 129.3 127.6 127.6
83.9 77.4 77.2 76.9 76.2 74.0 72.8
42.7
21.0 20.9 17.5 17.5
AcO
OAc
Cl
f1 (ppm)

3e

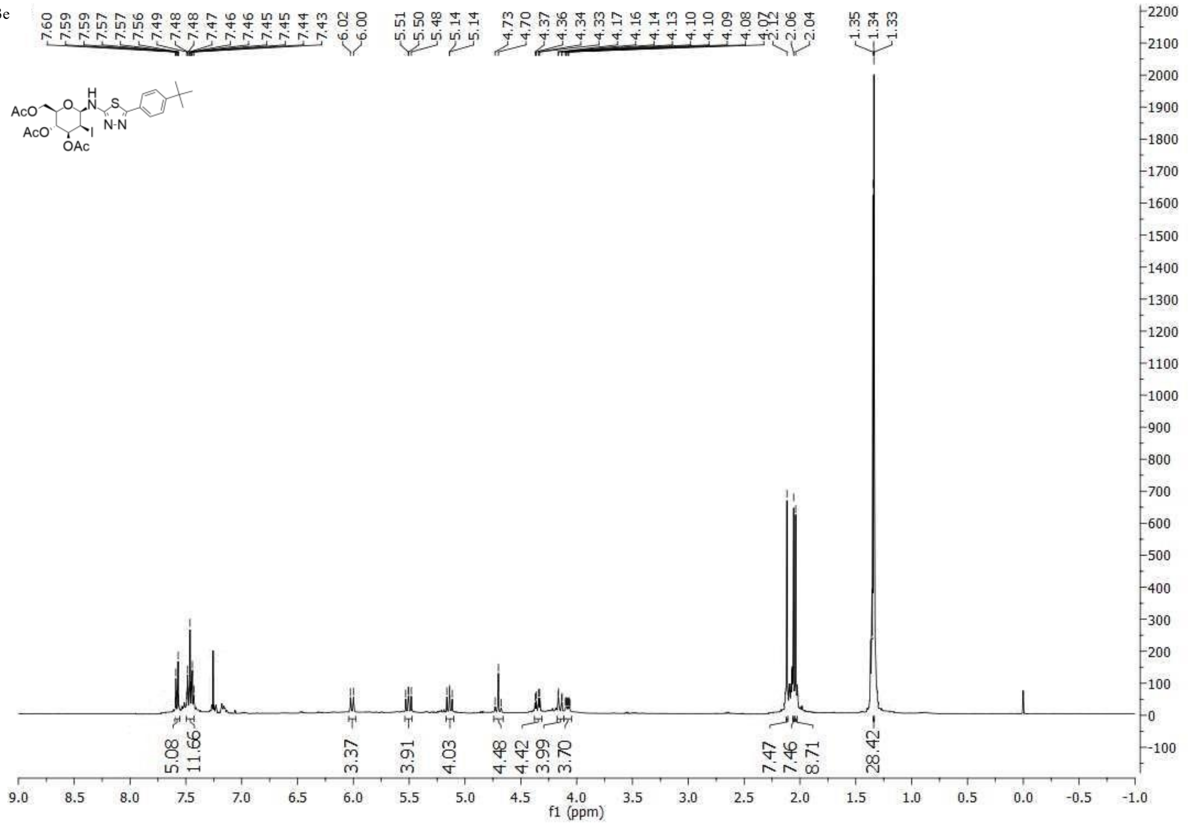

3f

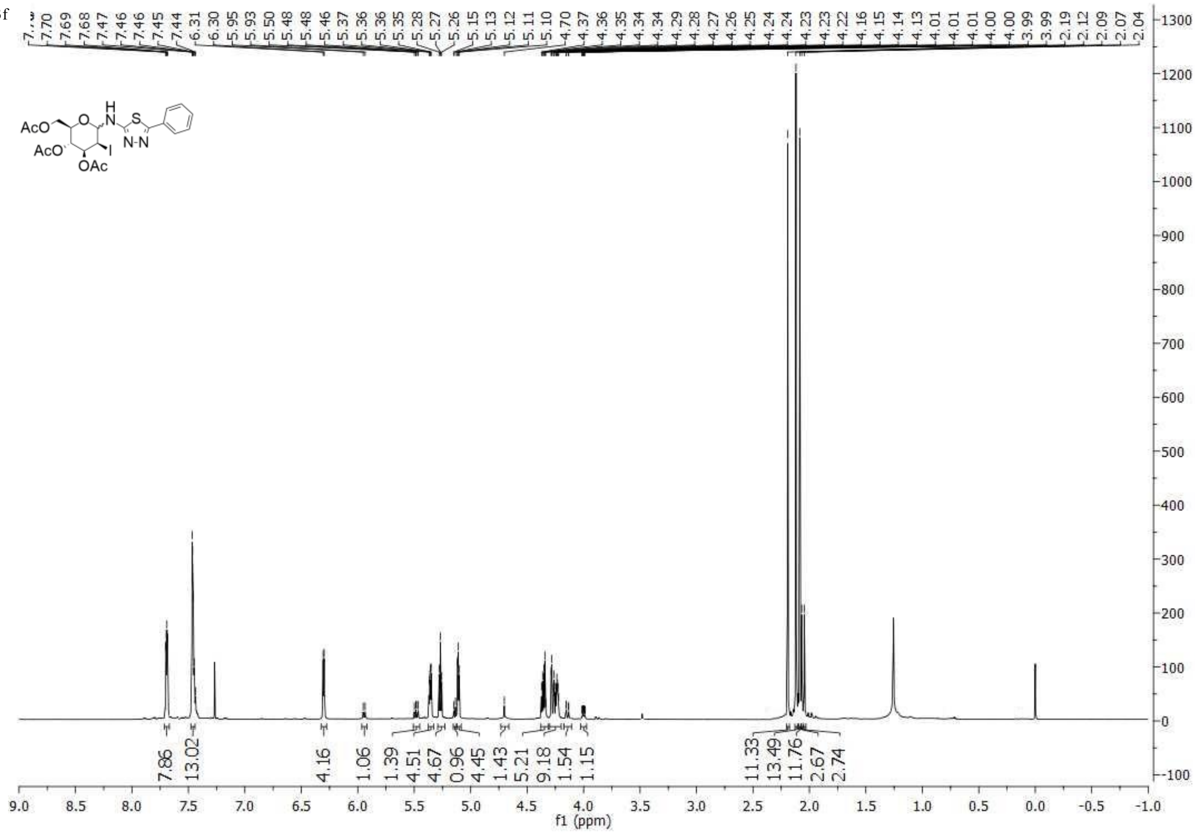

3f

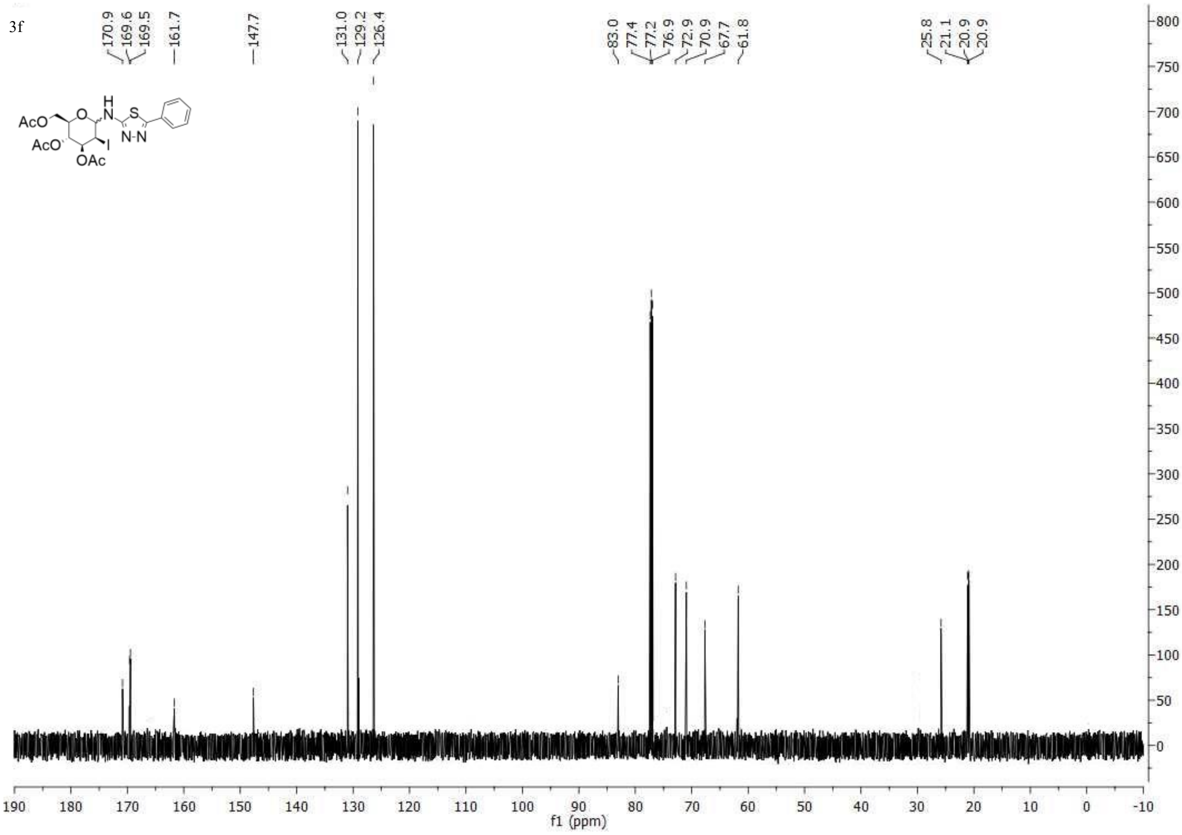

3g

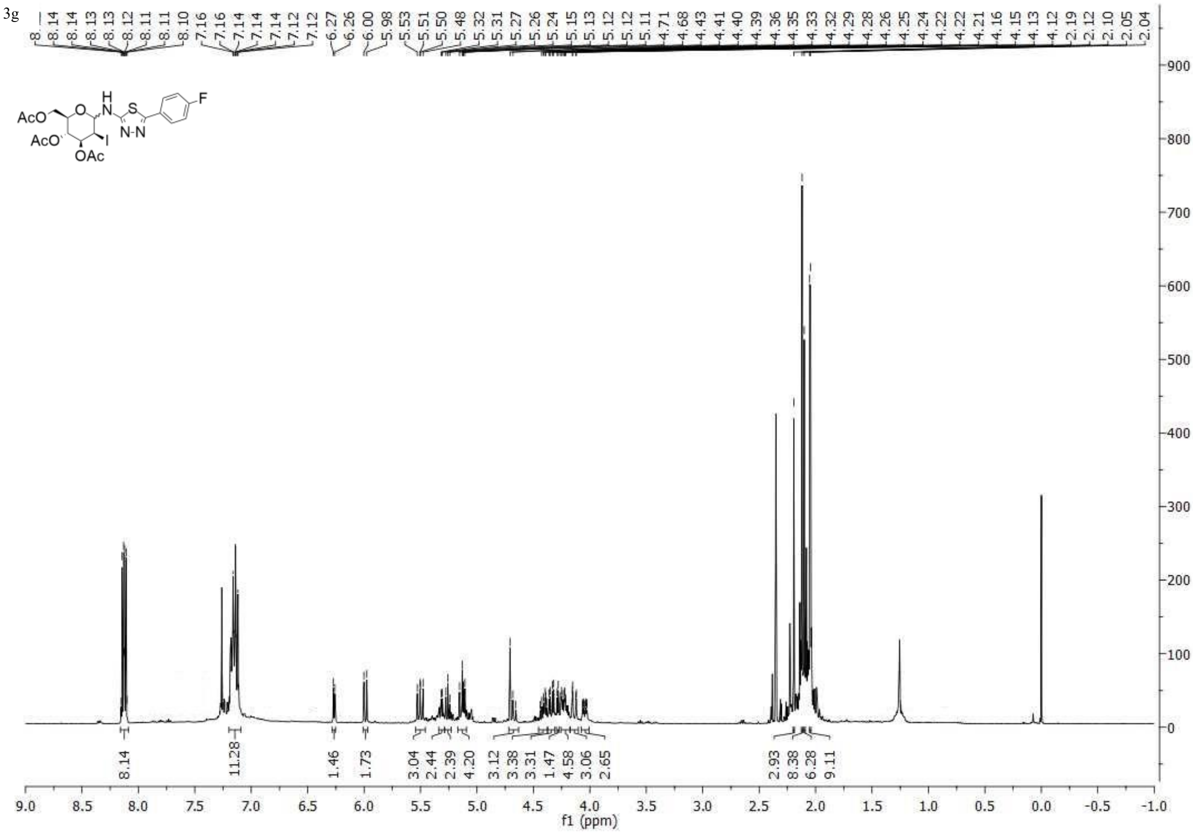

3g

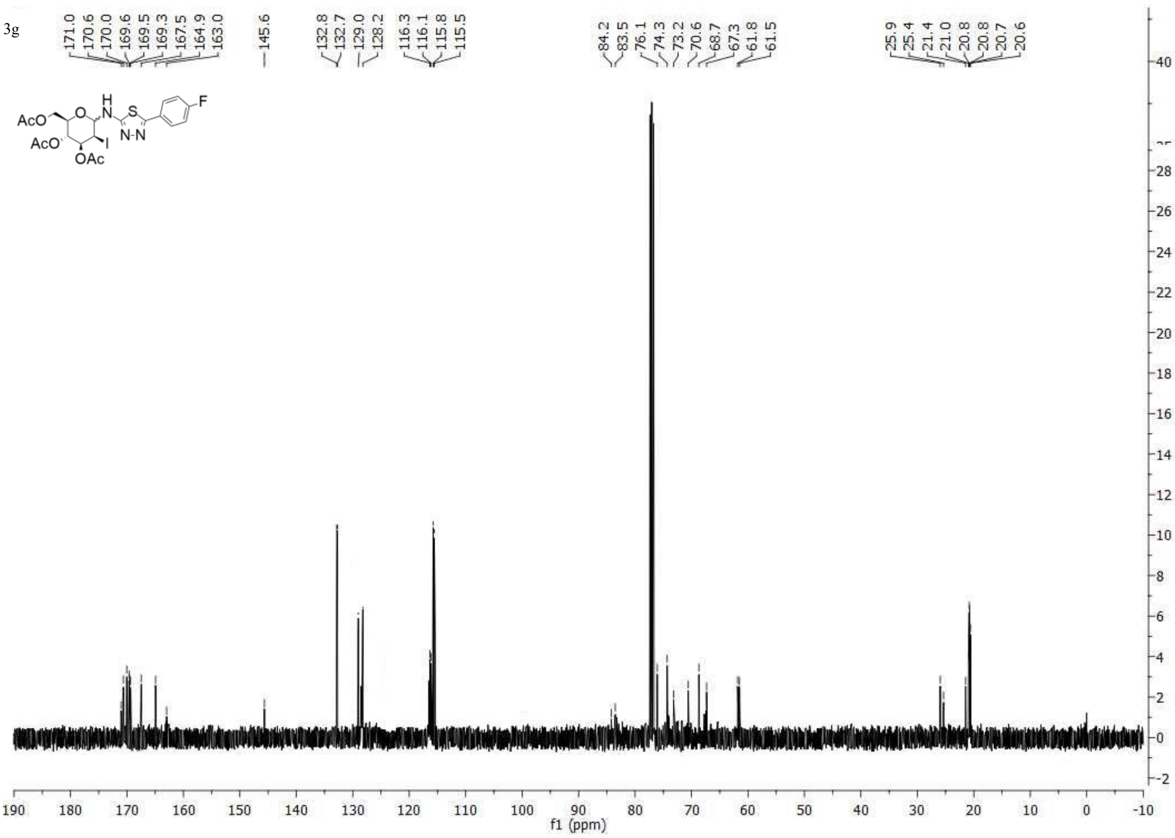

3h

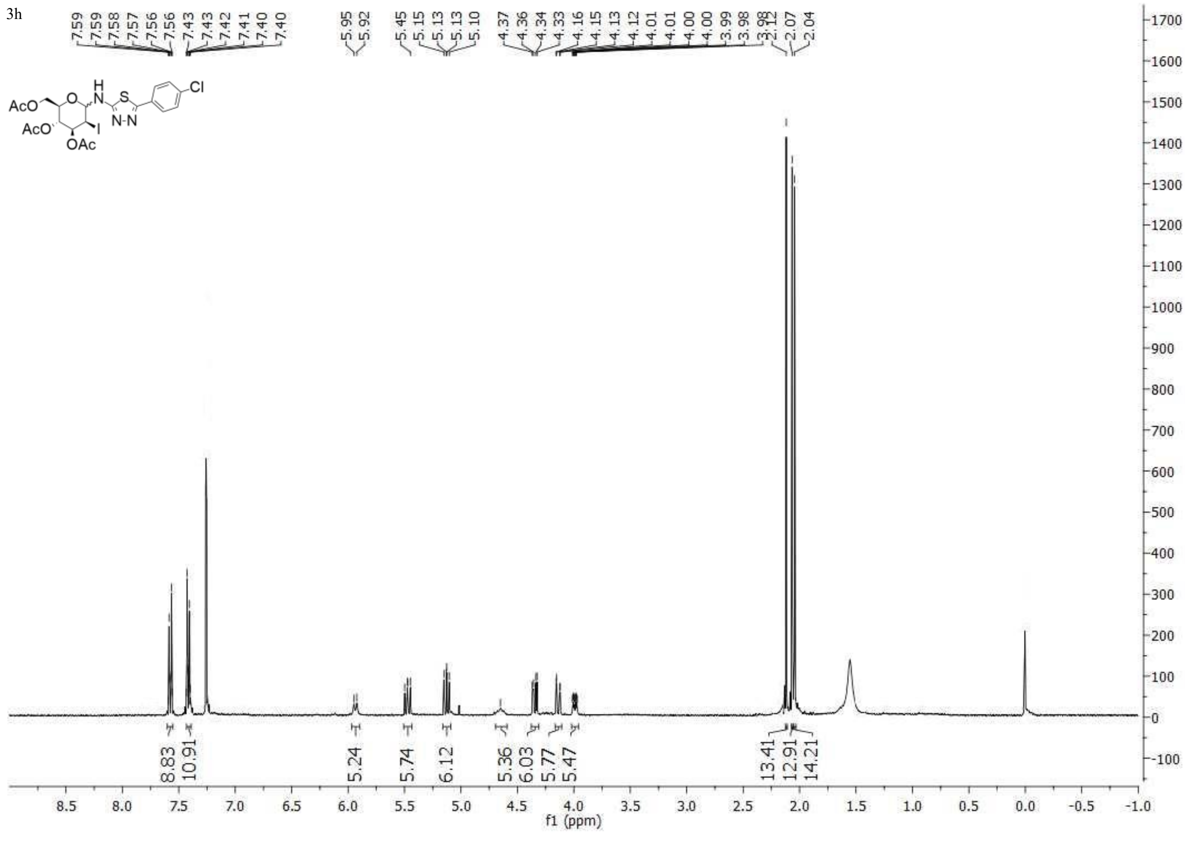

3i

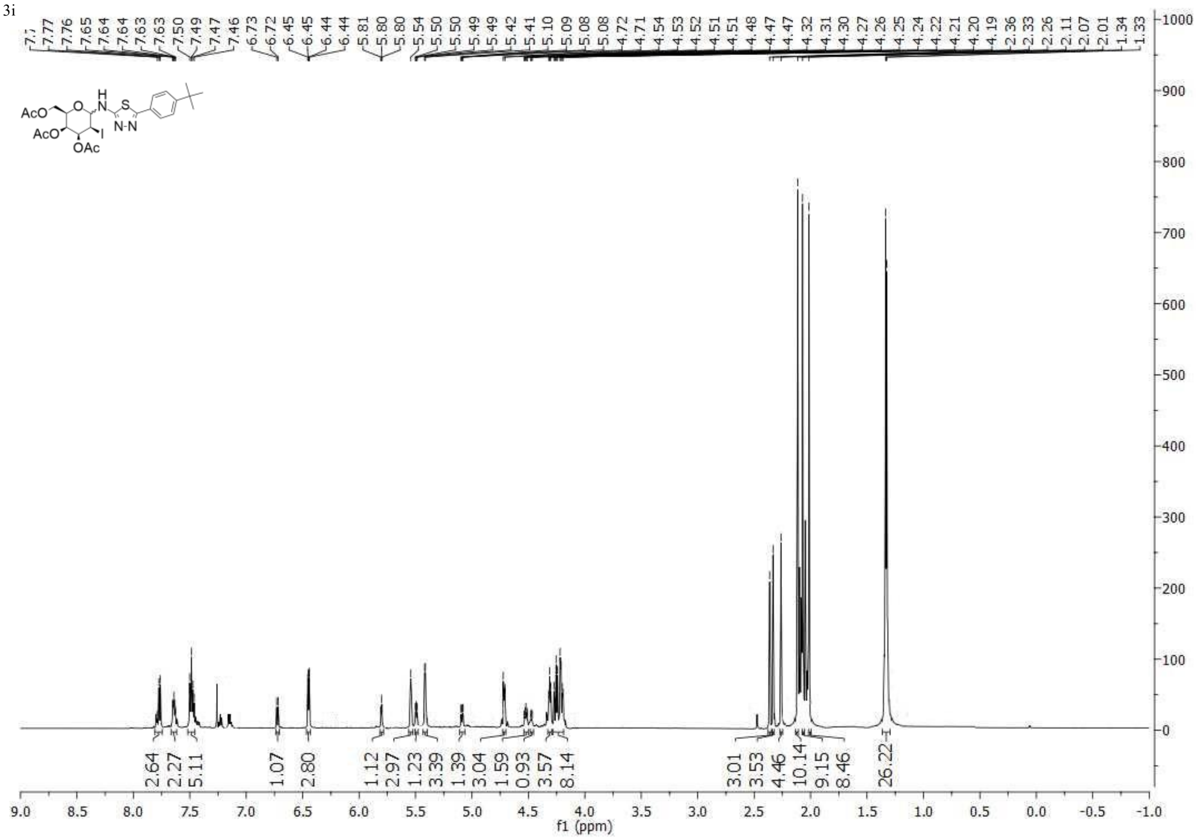

3i

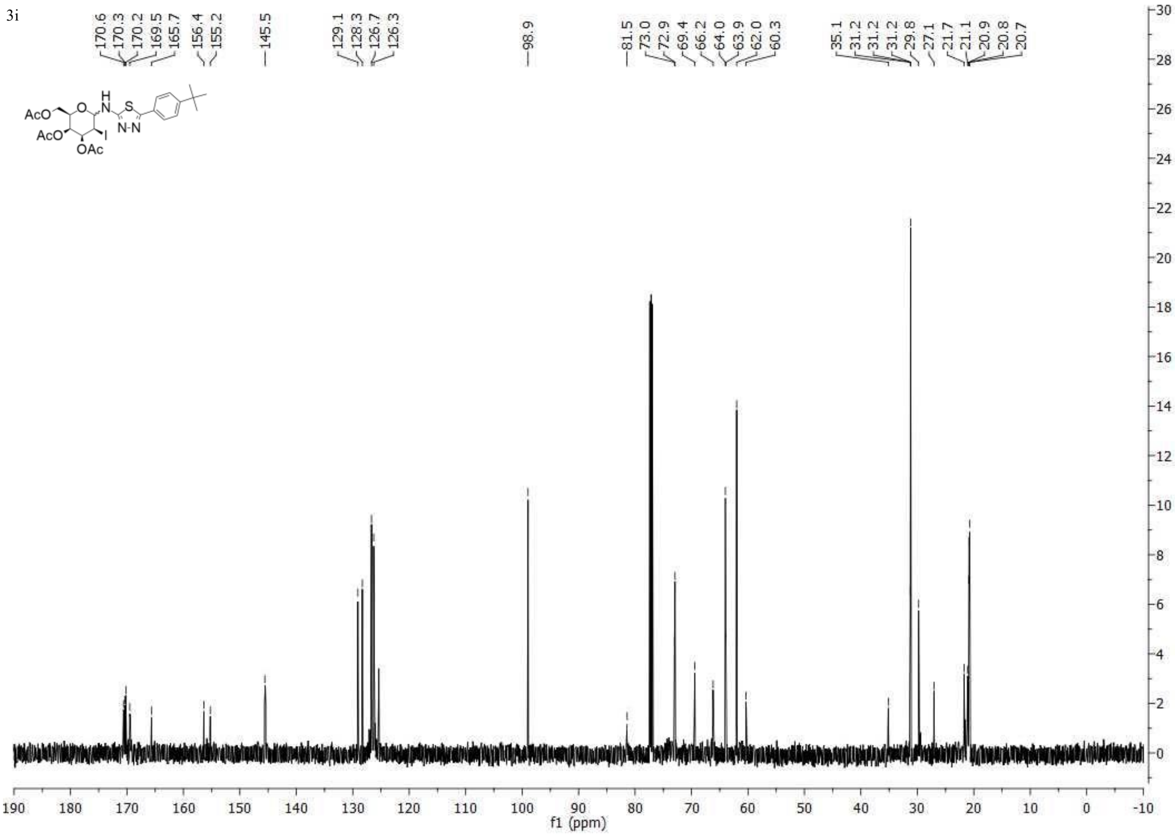

3j

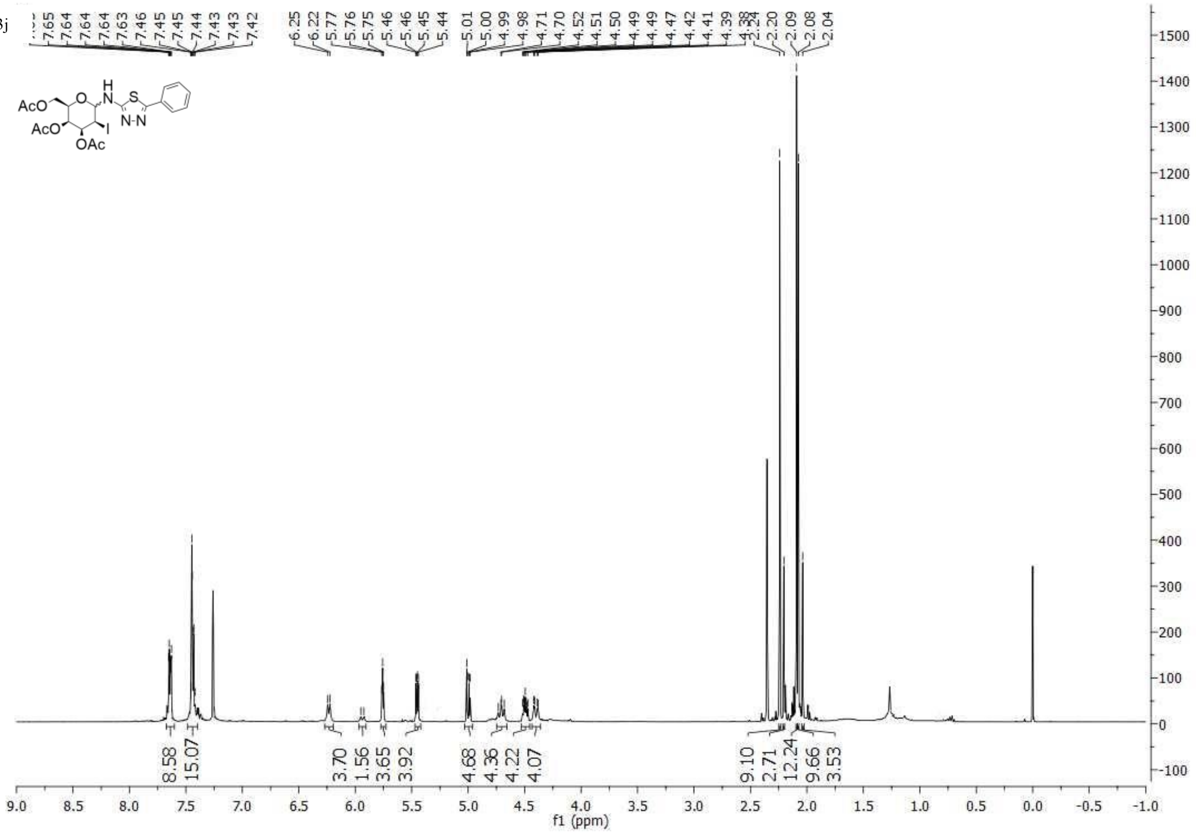

3k

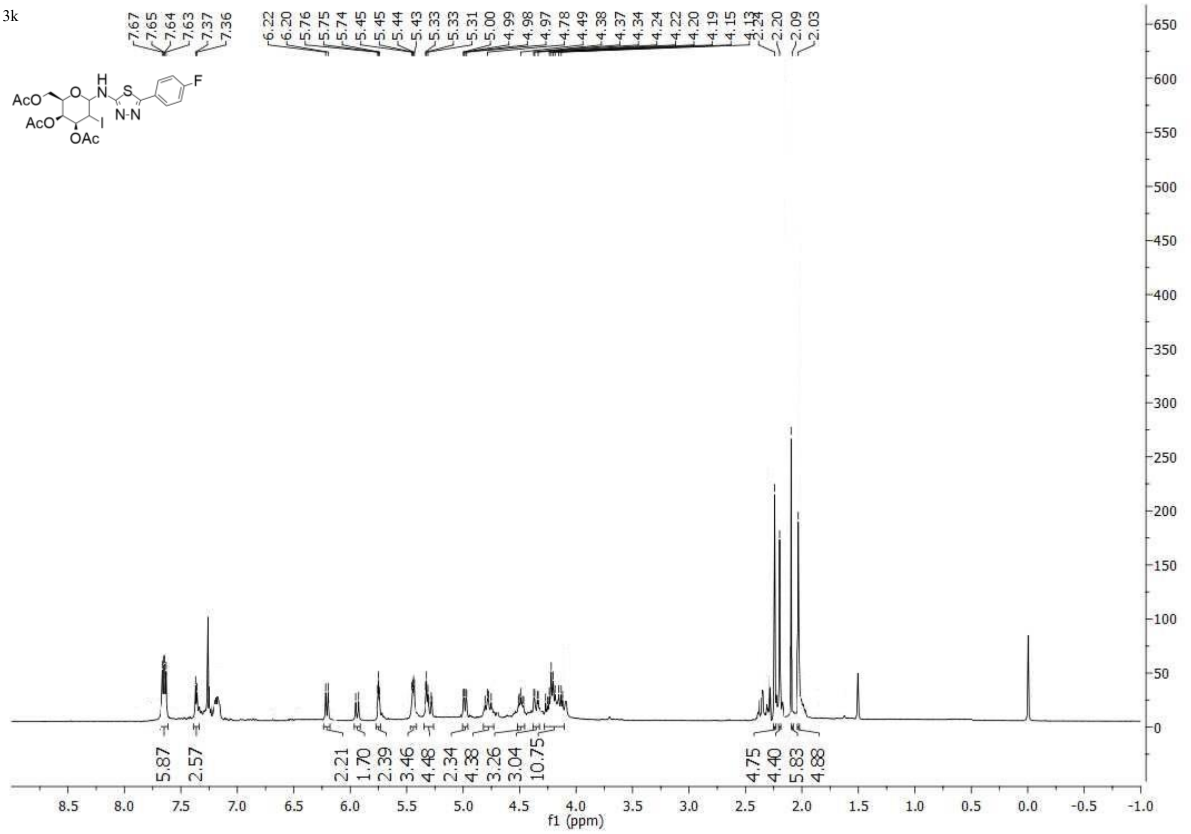

3l

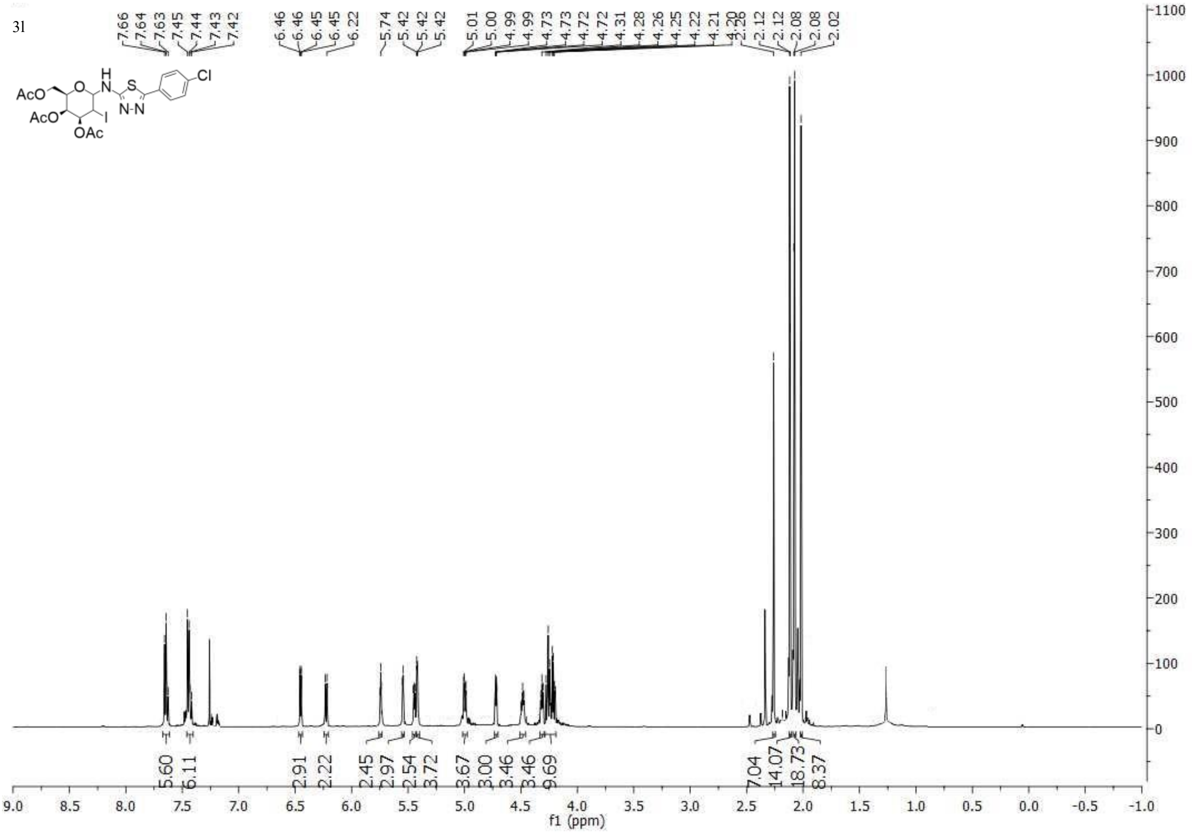
7.66 7.64 7.63 7.45 7.44 7.43 7.42 6.46 6.46 6.45 6.45 6.22 5.74 5.42 5.42 5.42 5.42 5.01 5.00 4.99 4.99 4.73 4.73 4.72 4.72 4.31 4.28 4.26 4.25 4.22 4.21 4.20 2.28 2.12 2.12 2.08 2.08 2.02
AcO
AcO
OAc
H
N
S
N-N
I
Cl
5.60 6.11 2.91 2.22 2.45 2.97 2.54 3.72 3.67 3.00 3.46 3.46 9.69 7.04 14.07 18.73 8.37
f1 (ppm)

5l

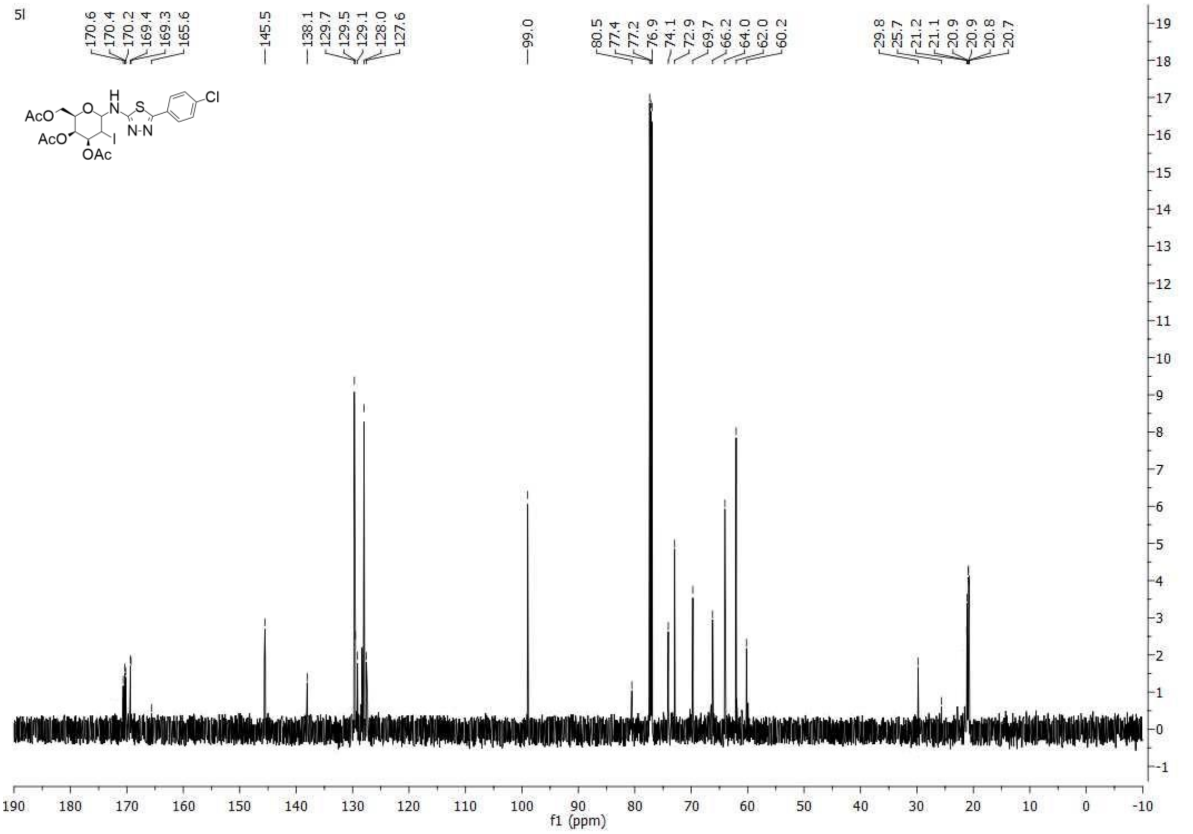
170.6 170.4 170.2 169.4 169.3 165.6 145.5 138.1 129.7 129.5 129.1 128.0 127.6 99.0 80.5 77.4 77.2 76.9 74.1 72.9 69.7 66.2 64.0 62.0 60.2 29.8 25.7 21.2 21.1 20.9 20.9 20.8 20.7
AcO
AcO
OAc
H
N
S
N-N
I
Cl
f1 (ppm)

3m

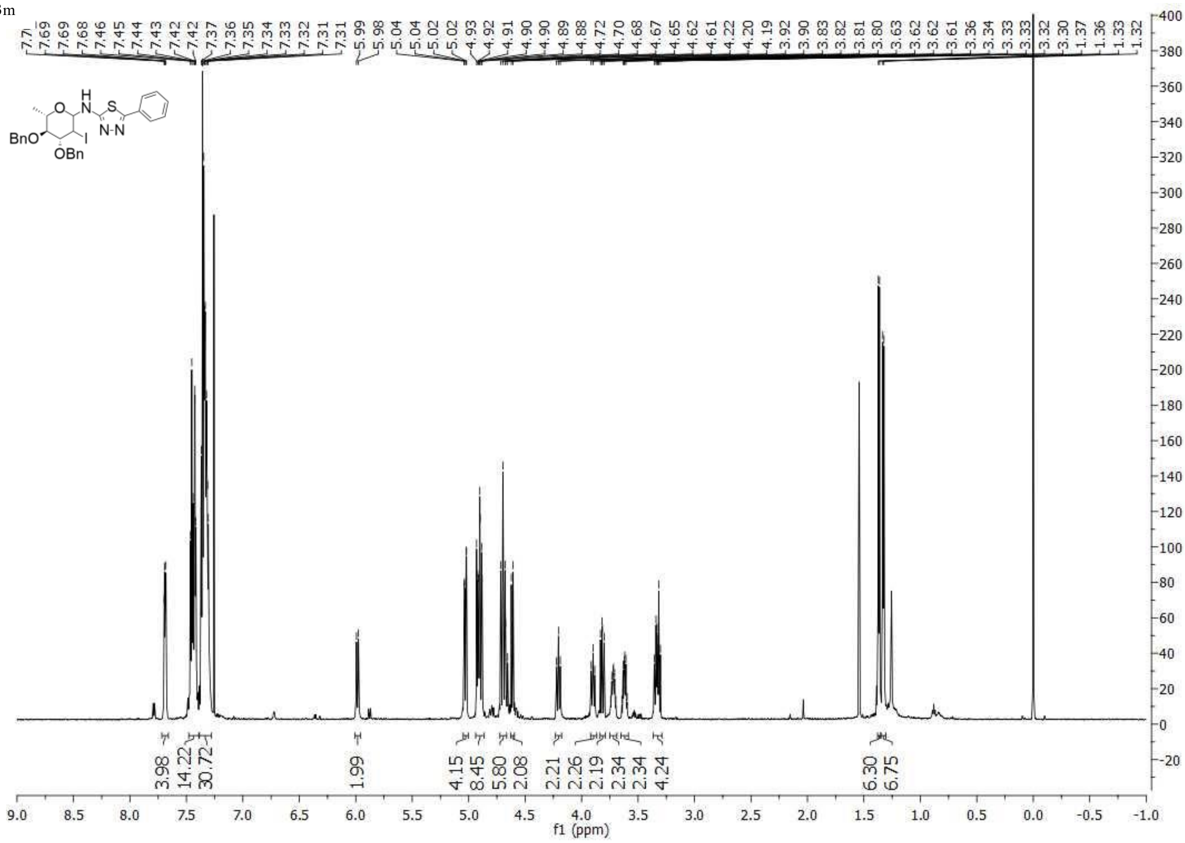

3m

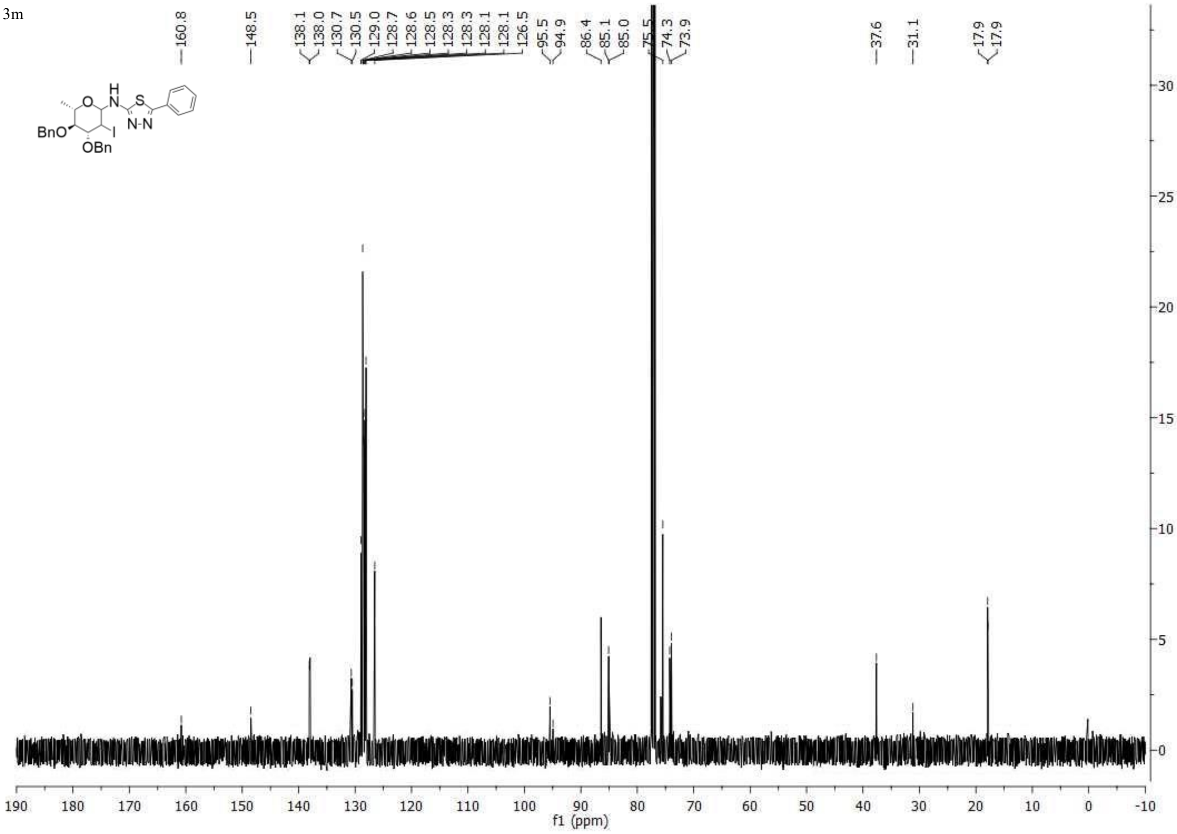

Figure 1. Typical histograms of PI stained DNA content during the cell cycle of MCF-7, HCT116, and HeLa cells, after 72 h of incubation with compounds at a dose of 100 µM. The statistical analysis was based on a t-test and a p-value less than 0.05 was considered statistically significant in comparison to the untreated controls (bolded ad colored).

MCF-7

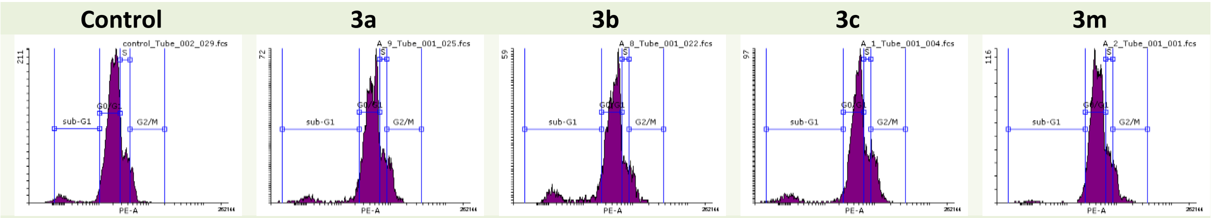

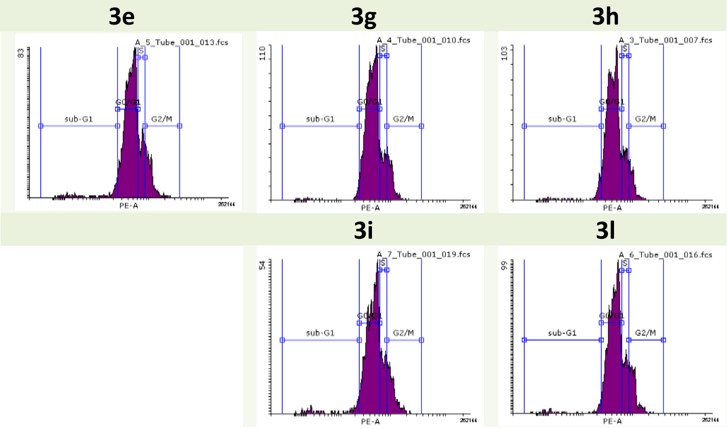

	T-test			
	p-value<0.05			
	sub-G1	G0/G1	S	G2/M
control	1	1	1	1
**3a**	**0,000103**	0,319706	**0,000352**	**0,040010**
**3b**	**0,002189**	0,212589	**0,024903**	0,960063
**3c**	**0,010566**	**0,000930**	0,075536	**0,000116**
**3m**	0,426771	**0,000759**	**0,011799**	**0,010788**
**3e**	0,260322	**0,031628**	0,502321	0,064252
**3g**	0,051476	**0,019460**	**0,022790**	0,583770
**3h**	0,218380	**0,041183**	**0,010312**	**0,004784**
**3i**	0,439154	0,357676	0,180332	**0,015997**
**3l**	0,158023	0,061884	0,242082	**0,000867**

HCT116

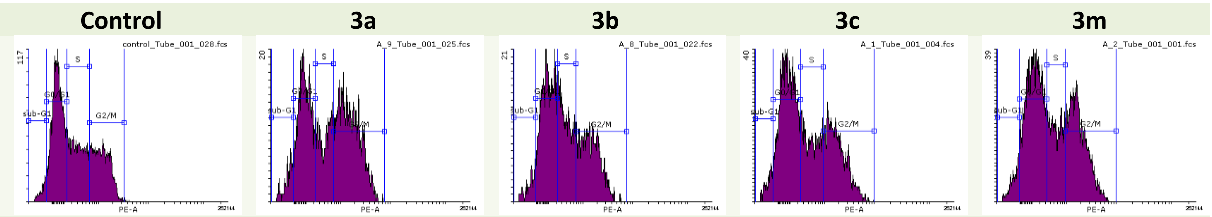

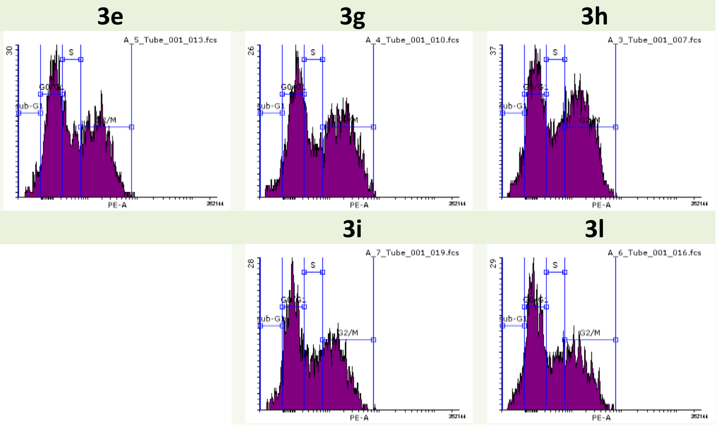

	T-test			
	p-value<0.05			
	sub-G1	G0/G1	S	G2/M
control	1	1	1	1
**3a**	**0,001131**	0,085751	**0,043034**	0,086691
**3b**	**0,001683**	**0,003769**	**0,000008**	**0,000172**
**3c**	**0,020129**	0,061965	**0,000483**	0,743282
**3m**	**0,000984**	0,163227	**0,000022**	**0,043993**
**3e**	0,094680	0,203904	**0,000107**	**0,008197**
**3g**	**0,010978**	**0,017721**	**0,000198**	**0,003061**
**3h**	**0,000366**	**0,009301**	**0,000020**	**0,000928**
**3i**	**0,001402**	0,165945	**0,000043**	**0,003860**
**3l**	**0,004817**	0,224124	**0,000027**	**0,032981**

HeLa

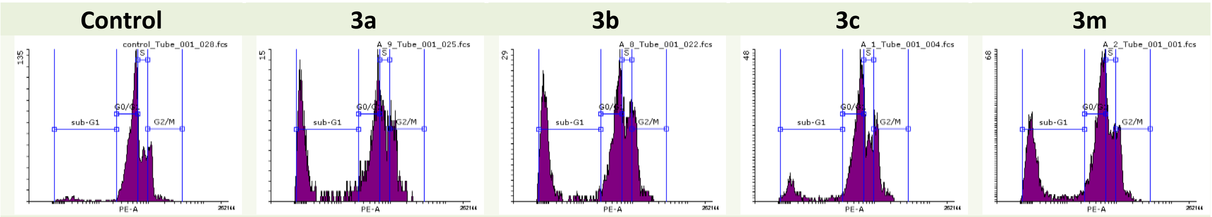

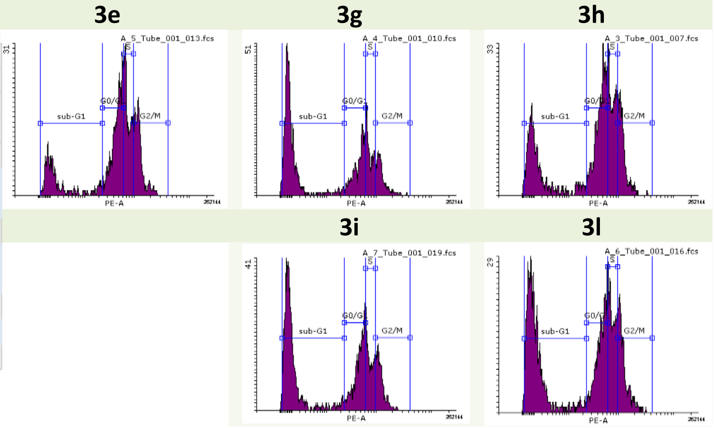

	T-test p-value<0.05			
	sub-G1	G0/G1	S	G2/M
control	1	1	1	1
**3a**	**0,000564**	**0,000078**	0,111111	0,711601
**3b**	**0,000050**	**0,000016**	0,297908	**0,005947**
**3c**	**0,000030**	**0,000214**	0,195897	**0,028271**
**3m**	**0,003147**	**0,000594**	0,137289	0,340180
**3e**	**0,000071**	**0,000029**	**0,001876**	**0,006987**
**3g**	**0,000002**	**0,000005**	**0,007686**	**0,006986**
**3h**	**0,002286**	**0,000255**	0,466604	0,234293
**3i**	**0,000004**	**0,000006**	**0,003328**	**0,020953**
**3l**	**0,002412**	**0,000077**	0,461925	0,054664

Figure 2. Typical dot plots of normal (Annexin-V negative and PI negative stained); early apoptotic (Annexin-V positive and PI negative stained); late apoptotic (Annexin-V positive and PI-positive stained); and necrotic (Annexin-V negative and PI-positive stained) MCF-7, HCT116, and HeLa cells after 72 h of incubation with compounds at a dose of 100 µM. The statistical analysis was based on a t-test and a p-value less than 0.05 was considered statistically significant in comparison to the untreated controls (bolded ad colored).

MCF-7

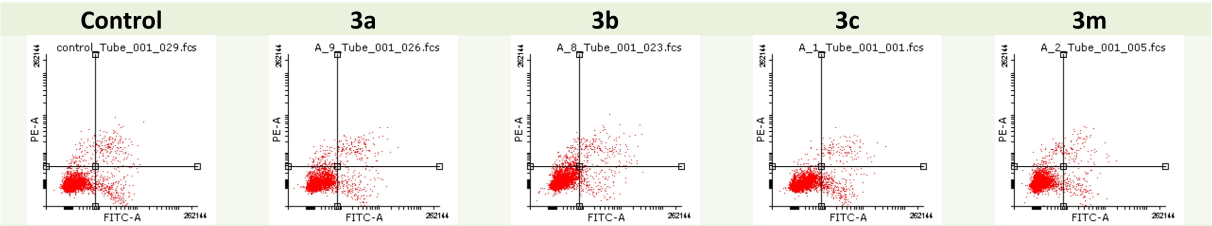

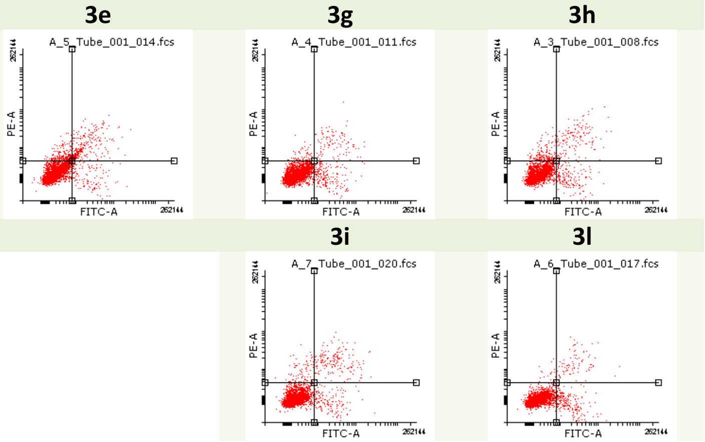

	T-test p-value<0.05			
	sub-G1	G0/G1	S	G2/M
control	1	1	1	1
**3a**	0,599478	0,413277	0,216650	**0,044658**
**3b**	0,285196	0,144580	0,314175	0,753437
**3c**	0,322616	0,050542	0,525614	0,065081
**3m**	0,078980	**0,039914**	0,094330	0,577868
**3e**	0,543781	0,133916	0,782403	0,072998
**3g**	0,293395	0,440890	0,225548	0,172249
**3h**	0,759250	**0,028541**	0,235240	**0,011945**
**3i**	0,138263	0,281611	0,150606	**0,019542**
**3l**	0,177454	**0,047062**	0,650490	0,451528

HCT116

Control | 3a | 3b | 3c | 3m

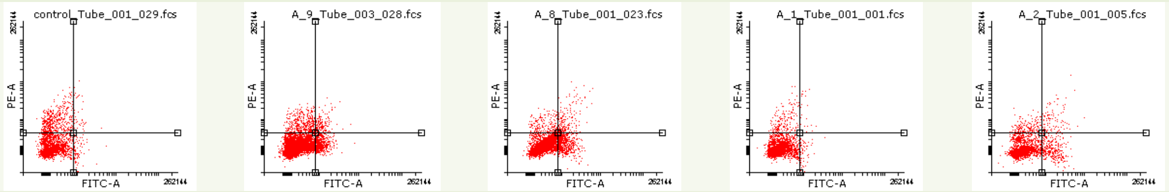

3e | 3g | 3h

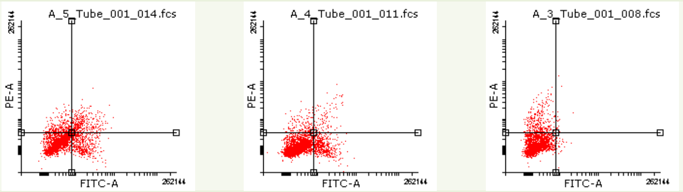

3i | 3l

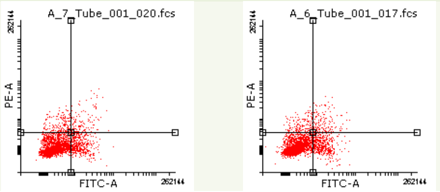

	T-test			
	p-value<0.05			
	sub-G1	G0/G1	S	G2/M
control	1	1	1	1
**3a**	**9,09838E-07**	0,172208	**0,004794**	**0,000005**
**3b**	**0,001890**	**0,000205**	**0,005343**	**0,000120**
**3c**	**0,000703**	**0,000026**	**0,008279**	**0,000004**
**3m**	0,281268	**0,000349**	**0,003678**	**0,000015**
**3e**	**0,000564**	**0,000220**	**0,003516**	**0,004503**
**3g**	0,361592	**0,000005**	**0,002602**	**0,000003**
**3h**	**0,000000**	**0,000008**	**0,000011**	**0,000188**
**3i**	**0,000106**	**0,000058**	**0,000042**	**0,000016**
**3l**	**0,000002**	**0,000002**	**0,000189**	**0,000004**

HeLa

Control | 3a | 3b | 3c | 3m

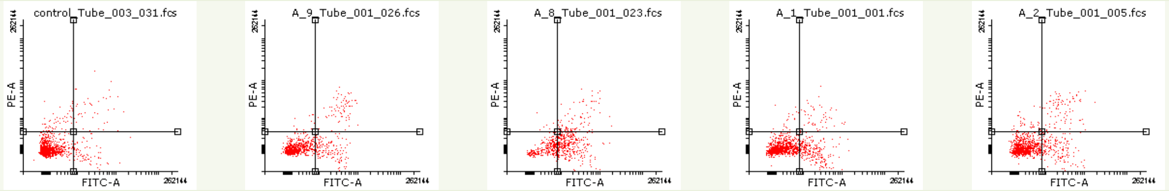

3e | 3g | 3h

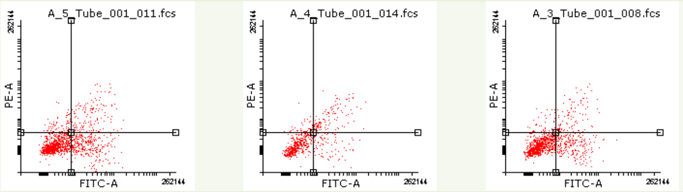

3i | 3l

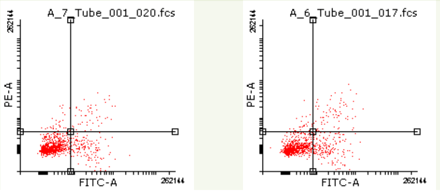

	T-test			
	p-value<0.05			
	sub-G1	G0/G1	S	G2/M
control	1	1	1	1
**3a**	**4,76E-05**	**0,000018**	0,219070	**0,000190**
**3b**	**0,183353**	**0,456644**	**0,010480**	**0,081834**
**3c**	0,000028	0,000717	**0,004741**	0,000186
**3m**	**0,000590**	**0,000092**	**0,002925**	**0,059722**
**3e**	**0,000817**	**0,002990**	**0,001048**	0,271349
**3g**	**0,001178**	**0,001290**	**0,000205**	0,108669
**3h**	**0,000001**	**0,000111**	**0,009970**	0,504706
**3i**	**0,001283**	**0,000275**	0,059986	**0,041793**
**3l**	**0,000432**	**0,000709**	**0,030688**	0,264795
